# On Consensus-Optimality Trade-offs in Collaborative Deep Learning

**DOI:** 10.3389/frai.2021.573731

**Published:** 2021-09-14

**Authors:** Zhanhong Jiang, Aditya Balu, Chinmay Hegde, Soumik Sarkar

**Affiliations:** ^1^ Self-aware Complex Systems Lab, Department of Mechaical Engineering, Iowa State University, Ames, IA, Unitd States; ^2^ Tandon School of Engineering, New York University, New York, NY, United States

**Keywords:** distributed optimization, consensus-optimality, collaborative deep learning, sgd, convergence

## Abstract

In distributed machine learning, where agents collaboratively learn from diverse private data sets, there is a fundamental tension between *consensus* and *optimality*. In this paper, we build on recent algorithmic progresses in distributed deep learning to explore various consensus-optimality trade-offs over a fixed communication topology. First, we propose the *incremental consensus*-based distributed stochastic gradient descent (i-CDSGD) algorithm, which involves multiple consensus steps (where each agent communicates information with its neighbors) within each SGD iteration. Second, we propose the *generalized consensus*-based distributed SGD (g-CDSGD) algorithm that enables us to navigate the full spectrum from complete consensus (all agents agree) to complete disagreement (each agent converges to individual model parameters). We analytically establish convergence of the proposed algorithms for strongly convex and nonconvex objective functions; we also analyze the momentum variants of the algorithms for the strongly convex case. We support our algorithms via numerical experiments, and demonstrate significant improvements over existing methods for collaborative deep learning.

## 1 Introduction

### 1.1 Motivation

Scaling up deep learning algorithms in a distributed setting (Recht et al., 2011; LeCun et al., 2015; Jin et al., 2016) is becoming increasingly critical, impacting several applications such as learning in robotic networks (Lenz et al., 2015; Fang et al., 2019), the Internet of Things (IoT) (Gubbi et al., 2013; Lane et al., 2015; Hu et al., 2020), mobile device networks (Lane and Georgiev, 2015; Kang et al., 2020), and sensor networks (Ge et al., 2019; He et al., 2020). For instance, with the development of wireless communication and distributed computing technologies, intelligent sensor network has been emerging as a kind of large-scale distributed network systems, which request more advanced sensor fusion techniques that enable data privacy preservation (Jiang et al., 2017a; He et al., 2019), dynamic optimization (Yang et al., 2016), and intelligent learning (Tan, 2020). This paper aims at developing novel algorithms to facilitate collaborative deep learning in distributed settings such as distributed sensor networks (Lesser et al., 2012). Several distributed deep learning approaches have been proposed to address issues such as model parallelism (Dean et al., 2012), data parallelism (Dean et al., 2012; Jiang et al., 2017a), and the role of communication and computation (Li et al., 2014; Das et al., 2016).

**FIGURE 1:**
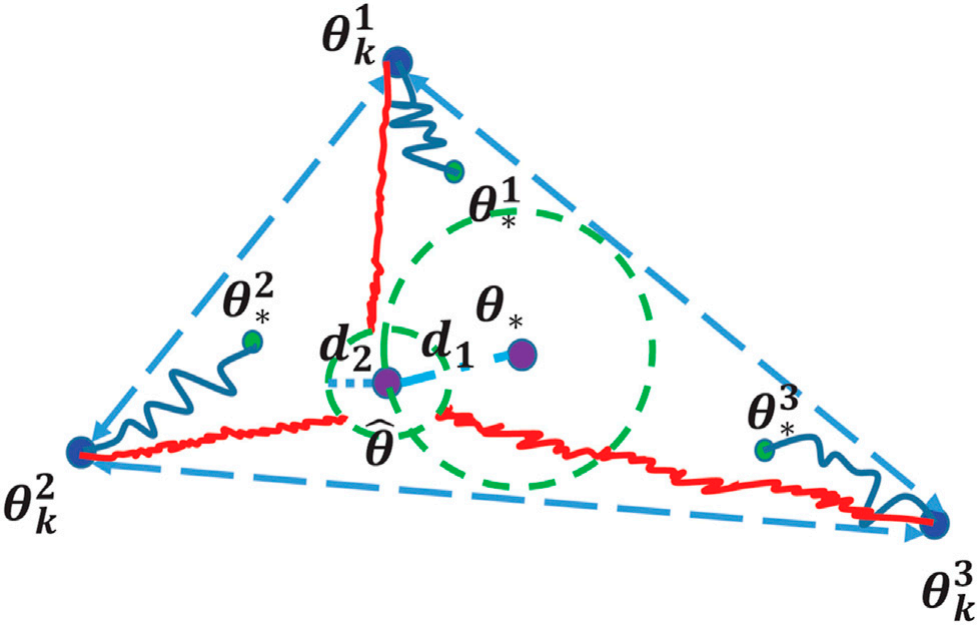
A closer look at the optimization updates in distributed deep learning: Blue dots represent the current states (i.e., learned model parameters) of the agents; green dots represent the individual local optima ($\theta_{*}^{i}$), that agents converge to without sufficient consensus; the purple dot (θ_*_) represents the ideal optimal point for the entire agent population; another purple dot $\hat{\theta }$ represents a possible consensus point for the agents which is far from optimal; blue and red curves signify the convergence trajectories with different step sizes; the green dashed circles indicate the neighborhoods of θ_*_ and $\hat{\theta }$, respectively; *d*_*2*_ represents the consensus bound/error and *d*_*1*_ represents the optimality bound/error; ideally, both of these bounds should be small.

We focus on the constrained communication topology setting where the data is distributed (so that each agent has its own estimate of the deep model) and where information exchange among the learning agents are constrained along the edges of a given communication graph (Jiang et al., 2017a; Lian et al., 2017). In this context, two key aspects arise: *consensus* and *optimality*. We refer the reader to Figure 1 for an illustration involving three agents. With sufficient information exchange, the learned model parameters corresponding to each agent, $\theta_{k}^{j},j=1,2,3$ could converge to $\hat{\theta }$, in which case they achieve consensus but not optimality (here, θ_*_ is the optimal model estimate if all the data were centralized). On the other hand, if no communication happens, the agents may approach their individual model estimates ($\theta_{*}^{i}$) while being far from consensus. The question is whether this trade-off between consensus and optimality can be balanced so that *all* agents collectively agree upon a model estimate close to θ_*_.

### 1.2 Our Contributions

In this paper, we propose, analyze, and empirically evaluate two new algorithmic frameworks for distributed deep learning that enable us to explore fundamental trade-offs between consensus and optimality. The first approach is called *incremental consensus*-based distributed stochastic gradient descent (i-CDSGD), which is a stochastic extension of the descent-style algorithm proposed in (Berahas et al., 2018). This involves running multiple consensus steps where each agent exchanges information with its neighbors within each SGD iteration. The second approach is called *generalized consensus*-based distributed SGD (g-CDSGD), based on the concept of generalized gossip (Jiang et al., 2017b). This involves a tuning parameter that explicitly controls the trade-off between consensus and optimality. Specifically, we:

•(**Algorithmic**) propose the i-CDSGD and g-CDSGD algorithms (along with their momentum variants).
•(**Theoretical**) prove the convergence of g-CDSGD (Theorems 1 and 3) and i-CDSGD (Theorems 2 and 4) for strongly convex and non-convex objective functions;
•(**Theoretical**) prove the convergence of the momentum variants of g-CDSGD (Theorem 5) and i-CDSGD (Theorem 6) for strongly convex objective functions;
•(**Practical**) empirically demonstrate that i-CDMSGD (the momentum variant of i-CDSGD) can achieve similar (global) accuracy as the state-of-the-art with lower fluctuation across epochs as well as better consensus;
•(**Practical**) empirically demonstrate that g-CDMSGD (the momentum variant of g-CDSGD) can achieve similar (global) accuracy as the state-of-the-art with lower fluctuation, smaller generalization error and better consensus.

We use both balanced and unbalanced datasets (i.e., equal or unequal distributions of training samples among the agents) for the numerical experiments with benchmark deep learning data sets. Please see Table 1 for a detailed comparison with existing algorithms.

**TABLE 1:** Comparisons between different optimization approaches.

Method	*F*	Con.Bou	Opt.Bou	Con.Rate	Mom.Ana	CC.T.	Sto
FedAvg McMahan et al. (2016)	Nonconvex	N/A	N/A	N/A	No	No	Yes
*DGD* ^*τ*^ Berahas et al. (2018)	Str-con	$O\left(\frac{\alpha }{1-\lambda_{2}^{\tau }}\right)$	$O\left(\frac{\alpha }{1-\lambda_{2}^{\tau }}\right)$	$O\left(\epsilon^{k}\right)$	No	Yes	No
MSDA Scaman et al. (2017)	Str-con	N/A	N/A	$O\left(\epsilon^{k}\right)$	Yes	Yes	No
	—	—	—	—	—	—	—
	—	—	—	—	—	—	—
CDSGD Jiang et al. (2017a)	Str-con	$O\left(\frac{\alpha }{1-\lambda_{2}}\right)$	$O\left(\frac{\alpha \gamma +1}{H+\alpha^{-1}\left(1-\lambda_{2}\right)}\right)$	$O\left(\epsilon^{k}\right)$	No	Yes	Yes
	Nonconvex	$O\left(\frac{\alpha }{1-\lambda_{2}}\right)$	$O\left(\alpha \gamma +1-\lambda_{N}\right)$	N/A			
	—	—	—	—	—	—	—
	—	—	—	—	—	—	—
Acc-DNGD-SC Qu and Li (2017)	Str-con	$O\left(\frac{\alpha^{\frac{1}{3}}}{\left(1-\lambda_{2}\right)\lambda_{2}^{\frac{2}{3}}}\right)$	N/A	$O\left(\epsilon^{k}\right)$	Yes	Yes	No
	—	—	—	—	—	—	—
	—	—	—	—	—	—	—
i-CDSGD [This paper]	Str-con	$O\left(\frac{\alpha }{1-\lambda_{2}^{\tau }}\right)$	$O\left(\frac{\alpha \gamma +1}{H+\alpha^{-1}\left(1-\lambda_{2}^{\tau }\right)}\right)$	$O\left(\epsilon^{k}\right)$	Yes	Yes	Yes
	Nonconvex	$O\left(\frac{\alpha }{1-\lambda_{2}^{\tau }}\right)$	Theorem 4	$O\left(k^{-1}\right)$	—	—	—
	—	—	—	—	—	—	—
	—	—	—	—	—	—	—
g-CDSGD [This paper]	Str-con	$O\left(\frac{\omega \alpha }{1-\lambda_{2}}\right)$	$O\left(\frac{\alpha \gamma -1+\omega^{-1}}{H}\right)$	$O\left(\epsilon^{k}\right)$	Yes	Yes	Yes
	Nonconvex	$O\left(\frac{\omega \alpha }{1-\lambda_{2}}\right)$	Theorem 3	$O\left(k^{-1}\right)$	—	—	—

Con.Bou.: consensus bound. Opt.Bou.: optimality bound. Con.Rate: convergence rate. Str-con: strongly convex. Mom.Ana.: momentum analysis. *α*: step size. *λ*_2_ ∈ (0, 1): the second largest eigen-value of a stochastic matrix. τ∈N: positive constant. *ω* ∈ (0, 1]: a positive constant. *ϵ* ∈ (0, 1): a positive constant, and it signifies the representative meaning. They are not exactly the same in different methods. Sto.: stochastic. C.C.T.: constrained communication topology. *c*_1_, *c*_2_ > 0: condition numbers. *H*: strong convexity constant. *γ* > 0: smoothness constant. The optimality bounds for i-CDSGD and g-CDSGD with nonconvex functions refer to the constant error bounds in Theorems 4 and 3.

### 1.3 Related Work

A large literature has emerged that studies distributed deep learning in both centralized and decentralized settings (Dean et al., 2012; Zhang et al., 2015; Blot et al., 2016; Jin et al., 2016; McMahan et al., 2016; Xu et al., 2017; Zhang et al., 2017; Zheng et al., 2017; Esfandiari et al., 2021), and we only attempt to summarize the most recent work (Wangni et al., 2017). proposed a gradient sparsification approach for communication-efficient distributed learning, while (Wen et al., 2017) proposed the concept of ternary gradients to reduce communication costs (Scaman et al., 2017). proposed a multi-step dual accelerated method using a gossip protocol to provide an optimal decentralized optimization algorithm for smooth and strongly convex loss functions. Decentralized parallel stochastic gradient descent (Lian et al., 2017) has also been proposed. In (Duchi et al., 2012), the authors developed a distributed averaging method for convex (possibly nonsmooth) objective functions; additionally (Mokhtari and Ribeiro, 2016), proposed a decentralized double stochastic averaging gradient algorithm. However, non-convex functions were not taken into account in either of the above works. Dual approaches (Uribe et al., 2020; Dvinskikh et al., 2019) were also proposed to address the convergence issues in the distributed optimization over networks while extra parameters need to be updated for obtaining the optimal solutions, which in return could increase the difficulty of solving the problem and the computational complexity. Again, convex problems were the main focus that might not enable the proposed schemes to generalize well for non-convex problems. Another category of approaches, the primal-dual gradient algorithms developed in (Hong et al., 2018; Dvinskikh and Gasnikov, 2019) were not evaluated by real-world datasets and were only originally specific for homogeneous networks where data was assumed independently identically distributed (i.i.d.).

Perhaps most closely related to this paper is the work of (Berahas et al., 2018), who presented a distributed optimization method (called *DGD* ^*τ*^) to enable consensus when the cost of communication is cheap. However, the authors only considered convex optimization problems, and only study deterministic gradient updates. Also (Qu and Li, 2017), proposed a class of (deterministic) accelerated distributed Nesterov gradient descent methods to achieve linear convergence rate, for the special case of strongly convex objective functions. In (Tsianos and Rabbat, 2012), both deterministic and stochastic distributed were discussed while the algorithm had no acceleration techniques. To our knowledge, none of these previous works have explicitly studied the trade-off between consensus and optimality. It should also be noted that the proposed approaches guarantee the convergence to the first-order stationary points for non-convex analysis and the avoidance of local maxima and saddle points is out of scope.

**Outline**: Section 2 presents the problem and several mathematical preliminaries. In Section 3, we present our two algorithmic frameworks, along with their analysis in Section 4. For validating the proposed schemes, several experimental results based on benchmark datasets are presented in Section 5. Concluding remarks are presented in Section 6.

## 2 Problem Formulation

We consider the standard unconstrained empirical risk minimization (ERM) problem typically used in machine learning problems (such as deep learning):

$$\text{min}\;\frac{1}{n}\overset{n}{\underset{i=1}{\sum }}f^{i}\left(\theta \right),$$ (1)

where $\theta \in R^{d}$ denotes the parameter vector of interest, $f:R^{d}\to R$ denotes a given loss function, and *f* ^*i*^ is the function value corresponding to a data point *i*. Our focus is to investigate the case where the ERM problem is solved collaboratively among a number of computational *agents*. In this paper, we are interested in problems where the agents exhibit *data parallelism*, i.e., they only have access to their own respective training datasets. However, we assume that the agents can communicate over a static undirected graph G=(V,E), where V is a vertex set (with nodes corresponding to agents) and E is an edge set. Throughout this paper we assume that the graph G is *connected*.

In this work, we primarily consider the spectrum between consensus and optimality and investigate thoroughly what effect such trade-offs have on the decentralized learning paradigm. Specifically, we analyze the theoretical properties of the proposed algorithms and show the empirical findings over benchmark datasets. However, in realistic scenarios, the graph may be subject to changes, such as the addition of new agents, and robust decentralized learning algorithms need to be developed for tackling such an issue, which is out of the scope and will definitely be one of our future research directions beyond this work.

Let $D_{j},\;j=1,\ldots ,n$ denote the subset of the training data (comprising *n*_*j*_ samples) corresponding to the *j* ^th^ agent such that $\sum_{j=1}^{N}n_{j}=n$, where *N* is the total number of agents. With this formulation, and since $f\left(\theta \right)=\sum_{j=1}^{N}f_{j}\left(\theta \right)$, we have the following (constrained) reformulation of Eq. 1:

$$\text{min}\;\overset{N}{\underset{j=1}{\sum }}\underset{i\in D_{j}}{\sum }f_{j}^{i}\left(\theta^{j}\right),\;\text{s.t.}\;\theta^{j}=\theta^{l}\;\;\forall \left(j,l\right)\in E,$$ (2)

Note that in this context, *θ* ^*j*^ for all *j* = 1, 2, … , *N* is the local copy of *θ*, which means the model architecture for each agent is typically the same. In another line of works where agents own different models, personalized federated/decentralized learning (Fallah et al., 2020) or meta-learning approaches (Fallah et al., 2021) have been developed correspondingly. Equivalently, the concatenated form of the above equation is as follows:

$$\text{min}\;F\left(\Theta \right)≔\overset{N}{\underset{j=1}{\sum }}\underset{i\in D_{j}}{\sum }f_{j}^{i}\left(\theta^{j}\right),\;\text{s.t.}\;\left(\Pi ⊗I_{d}\right)\Theta =\Theta ,$$ (3)

where $\Theta ≔{\left[\theta^{1};\theta^{2};\ldots ;\theta^{N}\right]}^{T}\in R^{dN}$, $\Pi \in R^{N\times N}$ is the agent interaction matrix with its entries *π*_*jl*_ indicating the link between agents *j* and *l*, *I*_*d*_ is the identity matrix of dimension *d* × *d*, and ⊗ represents the Kronecker product. Each element value in Π signifies the connection probability between two agents such that *π*_*jl*_ ∈ [0, 1]. One assumption is imposed for Π in the sequel to show the properties of any graph associated with the networked system.

We now introduce several key definitions and assumptions that characterize the above problem.

Definition 1.
*A function*$f:R^{d}\to R$*is said to beH-strongly convex, if for all*$x,y\in R^{d}$*, we have*$f\left(y\right)\geq f\left(x\right)+\nabla f{\left(x\right)}^{T}\left(y-x\right)+\frac{H}{2}\|y-x{\|}^{2}$*. It is said to beγ-smooth if*$f\left(y\right)\leq f\left(x\right)+\nabla f{\left(x\right)}^{T}\left(y-x\right)+\frac{\gamma }{2}\|y-x{\|}^{2}$*. Here,* ‖ ⋅‖ *represents the Euclidean norm.*

Definition 2.
*A functioncis said to be coercive if it satisfies:c*(*x*) → *∞ when* ‖*x*‖ → *∞* *.*

Assumption 1.
*The objective functions* $f_{j}:R^{d}\to R$ *are assumed to satisfy the following conditions: a) each* *f*_*j*_ *is* *γ*_*j*_ *-smooth; b) each* *f*_*j*_ *is proper (not everywhere infinite) and coercive; c) each* *f*_*j*_ *is Lipschitz continuous.*

Assumption 2.
*The interaction matrix* Π *is normalized to be doubly stochastic; the second largest eigenvalue of* Π *is strictly less than 1, i.e.,* *λ*_2_(Π)<1*, where* *λ*_2_(Π) *is the second largest eigenvalue of* Π*. If* (j,l)∉E *, then* *π*_*jl*_ = 0*.*

For convenience, we use *λ*_2_ to represent *λ*_2_(Π) and similar *λ*_*N*_ for *λ*_*N*_(Π), which signifies the *N*-largest eigenvalue of Π.

We will solve Eq. 2 in a distributed and stochastic manner.

For solving stochastic optimization problems, variants of the well-known stochastic gradient descent (SGD) have been commonly employed. For the formulation in Eq. 2, one of the state-of-the-art algorithms is a method called *consensus distributed SGD*, or CDSGD, recently proposed in (Jiang et al., 2017a). This method estimates *θ* according to the update equation:

$$\theta_{k+1}^{j}=\underset{l\in Nb\left(j\right)}{\sum }\pi_{jl}\theta_{k}^{l}-\alpha g_{j}\left(\theta_{k}^{j}\right)$$ (4)

where *Nb*(*j*) indicates the neighborhood of agent *j*, *α* is the step size, $g_{j}\left(\theta_{k}^{j}\right)$ is the (stochastic) gradient of *f*_*j*_ at $\theta_{k}^{j}$, implemented by drawing a minibatch of sampled data points. More precisely, $g_{j}\left(\theta_{k}^{j}\right)=\frac{1}{b^{\prime }}\sum_{q^{\prime }\in D^{\prime }}\nabla f_{j}^{q^{\prime }}\left(\theta_{k}^{j}\right)$, where *b*′ is the size of the minibatch $D^{\prime }$ selected uniformly at random from the data subset $D_{j}$ available to Agent *j*.

## 3 Proposed Algorithms

State-of-the-art algorithms such as CDSGD alternate between the *gradient update* and *consensus* steps. We propose two natural extensions where one can control the emphasis on *consensus* relative to the *gradient update* and hence, leads to interesting trade-offs between consensus and optimality.

### 3.1 Increasing Consensus

Observe that the concatenated form of the CDSGD updates, Eq. 4, can be expressed as

$$\Theta_{k+1}=\left(\Pi ⊗I_{d}\right)\Theta_{k}-\alpha g\left(\Theta_{k}\right),$$

If we perform *τ* consensus steps interlaced with each gradient update, we can obtain the following concatenated form of the iterations of the parameter estimates:

$$\Theta_{k+1}=\left(\Pi^{\tau }⊗I_{d}\right)\Theta_{k}-\alpha g\left(\Theta_{k}\right)$$ (5)

where, $g\left(\Theta_{k}\right)={\left[g_{1}^{T}\left(\theta_{k}^{1}\right),\;g_{2}^{T}\left(\theta_{k}^{2}\right),\ldots ,g_{N}^{T}\left(\theta_{k}^{N}\right)\right]}^{T}$. We call this variant *incremental* consensus-based distributed SGD (i-CDSGD) which is detailed in Algorithm 1. Note, in a distributed setting, that this algorithm incurs an additional factor *τ* in communication complexity.

In this context, i-CDSGD not only extends traditional decentralized (stochastic) gradient algorithms but also leverages the consensus among agents in a graph, particularly when agents are heterogeneous. Most traditional decentralized algorithms have been properly designed for homogeneous scenarios where agents share common properties such data sampling distributions and cannot be directly applied to heterogeneous networks as only one step of consensus may not be enough to enable agents to converge to the same solution due to the trade-off between the consensus-optimality. Therefore, the analysis presented in the paper for i-CDSGD provides a new perspective different from that most traditional decentralized algorithms delivered. A *different* and more direct approach to control the trade-off between consensus and gradient would be as follows:

$$\Theta_{k+1}=\left(1-\omega \right)\left(\Pi ⊗I_{d}\right)\Theta_{k}+\omega \left(\Theta_{k}-\alpha g\left(\Theta_{k}\right)\right)$$ (6)

where, 0<*ω* ≤ 1 is a user-defined parameter. We call this algorithm *generalized* consensus-based distributed SGD (g-CDSGD), and the full procedure is detailed in Algorithm 3.

Algorithm 1
Incremental Consensus-based Distributed Stochastic Gradient Descent

1: **Initialization**: $\theta_{0}^{j},v_{0}^{j},\;j=1,2,\ldots ,N$, *α*, *N*, *τ*, *m*, Π
2: Distribute the training data set to *N* agents
3: **for** *each agent* **do**
4: andomly shuffle each data subset
5: **for** *k* = 0: *m* **do**
6: *t* = 0
7: **for** *j* = 1, … , *N* **do**
8: $\theta_{t}^{j}=\theta_{k}^{j}${*Initialization before incremental consensus*}
9: **end for**
10: **while** *t* ≤ *τ* − 1 **do**
11: **for** *j* = 1, … , *N* **do**
12: $\theta_{t+1}^{j}=\sum_{l\in Nb\left(j\right)}\pi_{jl}\theta_{t}^{l}${*Incremental consensus*}
13: **end for**
14: *t* = *t* + 1
15: **end while**
16: $\hat{\theta }=\theta_{t}^{j}${*Update after incremental consensus*}
17: $\theta_{k+1}^{j}=\hat{\theta }-\alpha g_{j}\left(\theta_{k}^{j}\right)${*Update for iteration*}
18: **end for**
19: **end for**

Algorithm 2
Incremental Consensus-based Distributed Stochastic Gradient Descent w/ Momentum

1: **Initialization**: $\theta_{0}^{j},v_{0}^{j},\;j=1,2,\ldots ,N$, *α*, *N*, *τ*, *m*, Π, *μ*
2: Distribute the *Non-IID* training data set to *N* agents
3: **for** *each agent* **do**
4: Randomly shuffle each data subset
5: **for** *k* = 0: *m* **do**
6: *t* = 0
7: **for** *j* = 1, … , *N* **do**
8: $\theta_{t}^{j}=\theta_{k}^{j}${*Initialization before incremental consensus*}
9: $v_{t}^{j}=v_{k}^{j}${*Initialization of momentum before incremental consensus*}
10: **end for**
11: **while** *t* ≤ *τ* − 1 **do**
12: **for** *j* = 1, … , *N* **do**
13: $\theta_{t+1}^{j}=\sum_{l\in Nb\left(j\right)}\pi_{jl}\theta_{t}^{l}${*Incremental consensus for decision variable*}
14: $v_{t+1}^{j}=\sum_{l\in Nb\left(j\right)}\pi_{jl}v_{t}^{l}${*Incremental consensus for momentum*}
15: **end for**
16: *t* = *t* + 1
17: **end while**
18: $\hat{\theta }=\theta_{t}^{j}${*Update of decision variable after incremental consensus*}
19: $\hat{v}=v_{t}^{j}${*Update of momentum after incremental consensus*}
20: $v_{k+1}^{j}=\hat{\theta }-\theta_{k}^{j}+\mu \hat{v}-\alpha g_{j}\left(\theta_{k}^{j}\right)${*Update of momentum for iteration*}
21: $\theta_{k+1}^{j}=\theta_{k}^{j}+v_{k+1}^{j}${*Update of decision variable for iteration*}
22: **end for**
23: **end for**

By examining Eq. 6, we observe that when *ω* approaches 0, the update law boils down to a only consensus protocol, and that when *ω* approaches 1, the method reduces to standard stochastic gradient descent (for individual agents).

Next, we introduce the *Nesterov momentum* variants of our aforementioned algorithms. The momentum term is typically used for speeding up the convergence rate with high momentum constant close to Eq. 1 (Sutskever et al., 2013). More details can be found in Algorithms 2 and 4.

Algorithm 3
Generalized Consensus-based Distributed Stochastic Gradient Descent

1: **Initialization**: *ω*, $\theta_{0}^{j},v_{0}^{j},\;j=1,2,\ldots ,N$, *α*, *N*, *m*, Π
2: Distribute the training data set to *N* agents
3: **for** *each agent* **do**
4: Randomly shuffle each data subset
5: **for** *k* = 0: *m* **do**
6: $\hat{\theta }=\sum_{l\in Nb\left(j\right)}\pi_{jl}\theta_{k}^{l}${*Consensus update for decision variable only*}
7: $\theta_{k+1}^{j}=\left(1-\omega \right)\hat{\theta }+\omega \left(\theta_{k}^{j}-\alpha g_{j}\left(\theta_{k}^{j}\right)\right)${*Generalized consensus*}
8: **end for**
9: **end for**

We provide a discussion on the trade-off between the consensus and optimality to conclude this section. The trade-off between consensus and optimality can vary from convex to non-convex optimization problems. For most convex distributed optimization problems, they are well defined and globally optimal solution are not empty (probably unique if strongly convex) so each agent can communicate with other agents in its neighborhood to reach consensus and their local gradient updates will guide them to an minimizer. Therefore, the trade-off can be perfectly balanced to get to good optimal solutions. However, for non-convex problems, there exist possibly numerous locally optimal solutions such that the trade-off plays a critical role in the distributed optimization. The consensus among agents may not be necessarily a good optimal solution since the local gradient update of an agent may “dominate” the solution searching process and allows for “bias”. Hence, the investigation of such a trade-off is quite critical.

### 3.2 Tools for Convergence Analysis

We now analyze the convergence of the iterates $\left\{\theta_{k}^{j}\right\}$ generated by our algorithms. Specifically, we identify an appropriate Lyapunov function (that is bounded from below) for each algorithm that decreases with each iteration, thereby establishing convergence.

Algorithm 4
Generalized Consensus-based Distributed Stochastic Gradient Descent w/ Momentum

1: **Initialization**: *ω*, $\theta_{0}^{j},v_{0}^{j},\;j=1,2,\ldots ,N$, *α*, *N*, *m*, Π, *μ*
2: Distribute the *Non-IID* training data set to *N* agents
3: **for** *each agent* **do**
4: Randomly shuffle each data subset
5: **for** *k* = 0: *m* do
6: $\hat{\theta }=\sum_{l\in Nb\left(j\right)}\pi_{jl}\theta_{t}^{l}${*Consensus update for decision variable*}
7: $\hat{v}=\sum_{l\in Nb\left(j\right)}\pi_{jl}v_{t}^{l}${*Consensus update for momentum*}
8: $v_{k+1}^{j}=\left(1-\omega \right)\left(\hat{\theta }-\theta_{k}^{j}+\mu \hat{v}\right)+\omega \mu v_{k}^{j}-\omega \alpha g_{j}\left(\theta_{k}^{j}+\mu v_{k}^{j}\right)${*Generalized consensus for momentum*}
9: $\theta_{k+1}^{j}=\theta_{k}^{j}+v_{k+1}^{j}${*Update of decision variable for iteration*}
10: end for
11: end for

In our analysis, we use the concatenated (Kronecker) form of the updates. For simplicity, let $P=\Pi ⊗I_{d}\in R^{Nd\times Nd}$.

We begin the analysis for g-CDSGD by constructing a Lyapunov function that combines the true objective function with a regularization term involving a quadratic form of consensus as follows:

$$V\left(\Theta \right)≔\omega F\left(\Theta \right)+\frac{1-\omega }{2\alpha }\Theta^{T}\left(I_{Nd}-P\right)\Theta$$ (7)

It is easy to show that $\sum_{j=1}^{N}f_{j}\left(\theta^{j}\right)$ is *γ*_*m*_≔max_*j*_{*γ*_*j*_}-smooth, and that *V*(*Θ*) is $\hat{\gamma }$-smooth with

$$\hat{\gamma }≔\omega \gamma_{m}+\left(1-\omega \right)\alpha^{-1}\lambda_{\text{max}}\left(I_{Nd}-P\right)=\omega \gamma_{m}+\left(1-\omega \right)\alpha^{-1}\left(1-\lambda_{N}\right).$$

Likewise, it is easy to show that $\sum_{j=1}^{N}f_{j}\left(\theta^{j}\right)$ is *H*_*m*_≔  min_*j*_{*H*_*j*_}-strongly convex; therefore *V*(*Θ*) is $\hat{H}$-strongly convex with

$$\hat{H}≔\omega H_{m}+\left(1-\omega \right){\left(2\alpha \right)}^{-1}\lambda_{\text{min}}\left(I_{Nd}-P\right)=\omega H_{m}+\left(1-\omega \right){\left(2\alpha \right)}^{-1}\left(1-\lambda_{2}\right).$$

We also assume that there exists a lower bound *V*_inf_ for the function value sequence {*V* (Θ_*k*_)}, *∀k*. When the objective functions are strongly convex, we have *V*_inf_ = *V* (Θ*), where Θ* is the optimizer. Due to Assumptions 1 and 2, it is straightforward to obtain an equivalence between the gradient of Eq. 7 and the update law of g-CDSGD. Rewriting (6), we get:

$$\Theta_{k+1}=\left(1-\omega \right)P\Theta_{k}+\omega \left(\Theta_{k}-\alpha g\left(\Theta_{k}\right)\right)$$ (8)

Therefore, we obtain:

$$\begin{array}{cc} \Theta_{k+1} & =\Theta_{k}-\Theta_{k}+\left(1-\omega \right)P\Theta_{k}+\omega \left(\Theta_{k}-\alpha g\left(\Theta_{k}\right)\right)\\ & =\Theta_{k}-\alpha \omega \Theta_{k}-\left(1-\omega \right)I_{Nd}\Theta_{k}+\left(1-\omega \right)P\Theta_{k}\\ & =\Theta_{k}-\alpha \underset{\text{Lyapunov Gradient}}{\underset{︸}{\left(\omega g\left(\Theta_{k}\right)+\frac{1}{\alpha }\left(1-\omega \right)\left(I_{Nd}-P\right)\Theta_{k}\right)}} \end{array}$$ (9)

The last term in Eq. 9 is precisely the gradient of *V*(*Θ*). In the stochastic setting, g (Θ_*k*_) can be approximated by sampling one data point (or a mini-batch of data points) and the stochastic Lyapunov gradient is denoted by $S\left(\Theta_{k}\right),\forall k$.

Similarly, the update laws for our proposed Nesterov momentum variants can be compactly analyzed using the above Lyapunov function. First, we rewrite the updates for g-CDMSGD as follows:

$$\begin{array}{ll} & y_{k+1}=\Theta_{k}+\mu \left(\Theta_{k}-\Theta_{k-1}\right) \end{array}$$ (10a)

$$\begin{array}{ll} & \Theta_{k+1}=\left(1-\omega \right)Py_{k+1}+\omega \left(y_{k+1}-\alpha g\left(y_{k+1}\right)\right) \end{array}$$ (10b)

With a few algebraic manipulations, we get:

$$\begin{array}{cc} \Theta_{k+1} & =y_{k+1}-y_{k+1}+\left(1-\omega \right)Py_{k+1}+\omega \left(y_{k+1}-\alpha g\left(y_{k+1}\right)\right)\\ & =y_{k+1}-\alpha \left(\omega g\left(y_{k+1}\right)+\frac{1-\omega }{\alpha }\left(I_{Nd}-P\right)y_{k+1}\right) \end{array}$$ (11)

The above derivation simplifies the Nesterov momentum-based updates into a regular form which is more convenient for convergence analysis. For clarity, we separate this into two sub-equations. Let $S\left(y_{k+1}\right)=\omega g\left(y_{k+1}\right)+\frac{1-\omega }{\alpha }\left(I_{Nd}-P\right)y_{k+1}$. Thus, the updates for g-CDMSGD can be expressed as

$$\begin{array}{ll} & y_{k+1}=\Theta_{k}+\mu \left(\Theta_{k}-\Theta_{k-1}\right) \end{array}$$ (12a)

$$\begin{array}{ll} & \Theta_{k+1}=y_{k+1}-\alpha S\left(y_{k+1}\right), \end{array}$$ (12b)

Please find the similar transformation for i-CDMSGD in Supplementary Section S1.

For analysis, we require a bound on the variance of the stochastic Lyapunov gradient $S\left(\Theta_{k}\right)$ such that the variance of the gradient noise^1^ can be bounded from above. The variance of $S\left(\Theta_{k}\right)$ is defined as:

$$Var\left[S\left(\Theta_{k}\right)\right]≔E\left[\|S\left(\Theta_{k}\right){\|}^{2}\right]-\|E\left[S\left(\Theta_{k}\right)\right]{\|}^{2}.$$

The following assumption is standard in SGD convergence analysis, and is based on Bottou et al. (2018).

Assumption 3.
*a) There exist scalarsr*_2_ ≥ *r*_1_>0 *such that* $\nabla V{\left(\Theta_{k}\right)}^{T}E\left[S\left(\Theta_{k}\right)\right]\geq r_{1}\|\nabla V\left(\Theta_{k}\right){\|}^{2}$ *and* $\left\|E\left[S\left(\Theta_{k}\right)\right]\|\leq r_{2}\|\nabla V\left(\Theta_{k}\right)\right\|$ *for all* k∈N *; b) There exist scalars* *B* ≥ 0 *and* *B*_*V*_ ≥ 0 *such that* $Var\left[S\left(\Theta_{k}\right)\right]\leq B+B_{V}\|\nabla V\left(\Theta_{k}\right){\|}^{2}$ *for all* k∈N *.*

Remark 1.
Assumption 3(a) guarantees sufficient descent of *V* in the direction of $-S\left(\Theta_{k}\right)$; Assumption 3(b) states that the variance of $S\left(\Theta_{k}\right)$ is bounded above by the second moment of ∇*V*(Θ_*k*_). The constant *B* can be regarded to represent the second moment of noise involving in the gradient $S\left(\Theta_{k}\right)$. Therefore, the second moment of $S\left(\Theta_{k}\right)$ can be bounded above as

$$E\left[\|S\left(\Theta_{k}\right){\|}^{2}\right]\leq B+B_{m}\|\nabla V\left(\Theta_{k}\right){\|}^{2},$$

where $B_{m}≔B_{V}+r_{2}^{2}\geq r_{1}^{2}\geq 0$. Note that this is slightly different from the conventional assumption in SGD analysis that the variance of stochastic gradients is bounded above by a single constant; in our context, we control the restriction of $S\left(\Theta_{k}\right)$ via two scalar constants. However, our analysis technique is otherwise similar.

For convergence analysis, we assume:

Assumption 4.
*There exists a constantG*>0 *such that*$\|\nabla V\left(x\right)\|\leq G,\forall x\in R^{dN}$*.*

We justify this assumption. As the Lyapunov function is a composite function with the true cost function which is Lipschitz continuous and the regularization term associated with consensus, it can be immediately obtained that ‖∇*V*(*x*)‖ is bounded above by some positive constant.

Before turning to our main results, we present two auxiliary technical lemmas.

Lemma 1.
*Let Assumptions* 1 *and* 2 *hold. The iterates of g-CDSGD (Algorithm* 3) *satisfy the following inequality* ∀k∈N *:*

$$E\left[V\left(\Theta_{k+1}\right)\right]-V\left(\Theta_{k}\right)\leq -\alpha \nabla V{\left(\Theta_{k}\right)}^{T}E\left[S\left(\Theta_{k}\right)\right]+\frac{\hat{\gamma }}{2}\alpha^{2}E\left[\|S\left(\Theta_{k}\right){\|}^{2}\right].$$ (13)

Lemma 2.
*Let Assumptions* 1*,* 2*, and* 3 *hold. The iterates of g-CDSGD (Algorithm* 3) *satisfy the following inequality* ∀k∈N *:*

$$E\left[V\left(\Theta_{k+1}\right)\right]-V\left(\Theta_{k}\right)\leq -\left(r_{1}-\frac{\hat{\gamma }}{2}\alpha B_{m}\right)\alpha \|\nabla V\left(\Theta_{k}\right){\|}^{2}+\frac{\hat{\gamma }}{2}\alpha^{2}B.$$ (14)

We provide the proof of Lemmas 1 and 2 in the Supplementary Section S1. To guarantee that the first term on the right hand side is strictly negative, the step size *α* should be chosen such that

$$0\leq \alpha \leq \frac{r_{1}-\left(1-\omega \right)\left(1-\lambda_{N}^{\tau }\right)B_{m}}{\omega B_{m}\gamma_{m}}.$$ (15)

## 4 Analysis and Main Results

This section presents the main results by analyzing the convergence properties of the proposed algorithms. Our main results are grouped as follows: 1) we provide rigorous convergence analysis for g-CDSGD and i-CDSGD for both strongly convex and non-convex objective functions. 2) we analyze their momentum variants only for strongly convex objective functions. It is noted that the proofs of theorems are provided in the main body while the proofs of lemmas and propositions are provided in the Supplementary Section S1.

### 4.1 Convergence Analysis for I-CDSGD and G-CDSGD

Our analysis will consist of two components: establishing an upper bound on how far away the estimates of the individual agents are with respect to their empirical mean (which we call the *consensus bound*), and establishing an upper bound on how far away the overall procedure is with respect to the optimum (which we call the *optimality bound*).

First, we obtain consensus bounds for the g-CDSGD and i-CDSGD as follows.

Proposition 1.
*(Consensus with fixed step size, g-CDSGD) Let Assumptions* 1*,* 2*,* 4 *hold. The iterates of g-CDSGD (Algorithm* 3) *satisfy the following inequality* ∀k∈N *, when* *α* *satisfies* Eq. 15 *,*

$$E\left[\left\|\theta_{k}^{j}-s_{k}\right\|\right]\leq \frac{\omega \alpha \sqrt{B+B_{m}G^{2}}}{1-{\hat{\lambda }}_{2}}$$ (16)

*where* $s_{k}=\frac{1}{N}\sum_{j=1}^{N}\theta_{k}^{j}$ *,* ${\hat{\lambda }}_{2}$ *is the second-largest eigenvalue of the matrix* **Q** = (1 − *ω*)**P** + *ωI*_*Nd*_ *.*

Proposition 2.
*(Consensus with fixed step size, i-CDSGD) Let Assumptions* 1*,* 2*,* 4 *hold. The iterates of i-CDSGD (Algorithm* 1) *satisfy the following inequality* ∀k∈N *, when* *α* *satisfies* $0\leq \alpha \leq \frac{r_{1}-\left(1-\lambda_{N}^{\tau }\right)B_{m}}{\gamma_{m}B_{m}}$ *,*

$$E\left[\left\|\theta_{k}^{j}-s_{k}\right\|\right]\leq \frac{\alpha \sqrt{B+B_{m}G^{2}}}{1-\lambda_{2}^{\tau }}$$ (17)

We provide a discussion on comparing the consensus bounds in the Supplementary Section S1. Next, we obtain optimality bounds for g-CDSGD and i-CDSGD.

Theorem 1.
(Convergence of g-CDSGD in strongly convex case) Let Assumptions 1, 2, and 3 hold. When the step size satisfies Eq. 15, the iterates of g-CDSGD (Algorithm 3) satisfy the following inequality ∀k∈N:

$$E\left[D_{k}\right]\leq C_{1}^{k-1}D_{1}+C_{2}\overset{k-1}{\underset{q=0}{\sum }}C_{1}^{q}$$ (18)

*where* $D_{k}=V\left(\Theta_{k}\right)-V^{*},C_{1}=1-\left(\omega \alpha H_{m}+\frac{1-\omega }{2}\left(1-\lambda_{2}\right)\right)r_{1},C_{2}=\frac{\left(\alpha^{2}\gamma_{m}\omega +\alpha \left(1-\omega \right)\left(1-\lambda_{N}\right)\right)B}{2}$ *.*

PROOF. Recalling Lemma 2 and using Definition 1 yield:

$$\begin{array}{cc} E & \left[V\left(\Theta_{k+1}\right)\right]-V\left(\Theta_{k}\right)\leq -(r_{1}-\frac{\hat{\gamma }}{2}\alpha B_{m})\alpha \|\nabla V\left(\Theta_{k}\right){\|}^{2}\\ & +\frac{\hat{\gamma }}{2}\alpha^{2}B\leq -\frac{1}{2}\alpha r_{1}\|\nabla \left(\Theta_{k}\right){\|}^{2}+\frac{\alpha^{2}\hat{\gamma }B}{2}\leq -\alpha r_{1}\hat{H}\left(V\left(\Theta_{k}\right)-V^{*}\right)+\frac{\alpha^{2}\hat{\gamma }B}{2}. \end{array}$$ (19)

The second inequality follows from that $\alpha \leq \frac{r_{1}}{\hat{\gamma }B_{m}}$, which is implied by Eq. 15. The expectation taken in the above inequalities is only related to *θ*_*k+1*_. Hence, recursively taking the expectation and subtracting V* from both sides, we get:

$$E\left[V\left(\Theta_{k+1}\right)-V^{*}\right]\leq \left(1-\alpha \hat{H}r_{1}\right)E\left[V\left(\Theta_{k}\right)-V^{*}\right]+\frac{\alpha^{2}\hat{\gamma }B}{2}.$$ (20)

As $0\leq \alpha \hat{H}r_{1}\leq \frac{\hat{H}r_{1}^{2}}{\hat{\gamma }B_{m}}\leq \frac{\hat{H}r_{1}^{2}}{\hat{\gamma }r_{1}^{2}}=\frac{\hat{H}}{\hat{\gamma }}\leq 1$, the conclusion follows by applying Eq. 1 recursively through iteration k∈N and letting $D_{k}=V\left(\Theta_{k}\right)-V^{*},C_{1}=1-\left(\omega \alpha H_{m}+\frac{1-\omega }{2}\left(1-\lambda_{2}\right)\right)r_{1},C_{2}=\frac{\left(\alpha^{2}\gamma_{m}\omega +\alpha \left(1-\omega \right)\left(1-\lambda_{N}\right)\right)B}{2}$.

Theorem 2.
(Convergence of i-CDSGD in strongly convex case) Let Assumptions 1, 2, and 3 hold. When the step size satisfies $0\leq \alpha \leq \frac{r_{1}-\left(1-\lambda_{N}^{\tau }\right)B_{m}}{\gamma_{m}B_{m}}$, the iterates of i-CDSGD (Algorithm 1) satisfy the following inequality ∀k∈N:

$$E\left[D_{k}\right]\leq C_{3}^{k-1}D_{1}+C_{4}\overset{k-1}{\underset{q=0}{\sum }}C_{3}^{q}$$ (21)

*where* *D*_*k*_ = *V* (Θ_*k*_) − V*, $C_{3}=1-\left(\alpha H_{m}+\frac{1}{2}\left(1-\lambda_{2}^{\tau }\right)\right)r_{1}$ *,* $C_{4}=\frac{\left(\alpha^{2}\gamma_{m}+\alpha \left(1-\lambda_{N}^{\tau }\right)\right)B}{2}$ *.*

PROOF. We omit the proof here and one can easily get it following the proof techniques shown for Theorem 1. The desired result is obtained by replacing *C*_1_ with *C*_3_ and *C*_2_ with *C*_4_, respectively.

Although we show the convergence for strongly convex objectives, we note that objective functions are highly non-convex for most deep learning applications. While convergence to a global minimum in such cases is extremely difficult to establish, we prove that g-CDSGD and i-CDSGD still exhibits weaker (but meaningful) notions of convergence.

Theorem 3.
*(Convergence to the first-order stationary point for non-convex case of g-CDSGD) Let Assumptions* 1*,* 2*, and* 3 *hold. When the step size satisfies* Eq. 15 *, the iterates of g-CDSGD (Algorithm* 3) *satisfy the following inequality* ∀K∈N *:*

$$E\left[\frac{1}{K}\overset{K}{\underset{k=1}{\sum }}\|\nabla V\left(\Theta_{k}\right){\|}^{2}\right]\leq \frac{\left(\omega \gamma_{m}\alpha +\left(1-\omega \right)\left(1-\lambda_{N}\right)\right)B}{r_{1}}+\frac{2\left(V\left(\Theta_{1}\right)-V_{\text{inf}}\right)}{Kr_{1}\alpha }$$ (22)

PROOF. Recalling Lemma 2 and taking expectations on both sides lead to the following relation:

$$E\left[V\left(\Theta_{k+1}\right)\right]-E\left[V\left(\Theta_{k}\right)\right]\leq -(r_{1}-\frac{\hat{\gamma }\alpha B_{m}}{2})\alpha E\left[\|\nabla V\left(\Theta_{k}\right){\|}^{2}\right]+\frac{\hat{\gamma }\alpha^{2}B}{2}.$$ (23)

If the step size is such that $\alpha \leq \frac{r_{1}}{\hat{\gamma }B_{m}}$, we get:

$$E\left[V\left(\Theta_{k+1}\right)\right]-E\left[V\left(\Theta_{k}\right)\right]\leq -\frac{r_{1}\alpha }{2}E\left[\|\nabla V\left(\Theta_{k}\right){\|}^{2}\right]+\frac{\alpha^{2}\hat{\gamma }B}{2}.$$ (24)

Applying the above inequality from 1 to *K* and summing them up can give the following relation

$$V_{\text{inf}}-V\left(\Theta_{1}\right)\leq E\left[V\left(\Theta_{k+1}\right)\right]-V\left(\Theta_{1}\right)\leq -\frac{r_{1}\alpha }{2}\overset{m}{\underset{k=1}{\sum }}E\left[\|\nabla V\left(\Theta_{k}\right){\|}^{2}\right]+\frac{m\alpha^{2}\hat{\gamma }B}{2}.$$ (25)

The last inequality follows from the Assumption 3. Rearrangement of the above inequality, substituting $\hat{\gamma }=\omega \gamma_{m}+\alpha^{-1}\left(1-\omega \right)\left(1-\lambda_{N}\right)$, and dividing by *K* yields the desired result.

Theorem 4.
*(Convergence to the first-order stationary point for non-convex case of i-CDSGD) Let Assumptions* 1*,* 2*, and* 3 *hold. When the step size satisfies* $0\leq \alpha \leq \frac{r_{1}-\left(1-\lambda_{N}^{\tau }\right)B_{m}}{\gamma_{m}B_{m}}$ *, the iterates of i-CDSGD (Algorithm* 1) *satisfy the following inequality* ∀K∈N *:*

$$E\left[\frac{1}{K}\overset{K}{\underset{k=1}{\sum }}\|\nabla V\left(\Theta_{k}\right){\|}^{2}\right]\leq \frac{\left(\gamma_{m}\alpha +\left(1-\lambda_{N}^{\tau }\right)\right)B}{r_{1}}+\frac{2\left(V\left(\Theta_{1}\right)-V_{\text{inf}}\right)}{Kr_{1}\alpha }.$$ (26)

PROOF. The proof for this theorem is rather similar to the one provided for Theorem 3 above, and we omit the details. □

Remark 2.
In the literature, to eliminate the negative effect of “noise” caused by the stochastic gradients, a diminishing step size is used. However, in our context, we observe from Theorems 1 and 2 that a constant step size itself can result in convergence, up to a neighborhood, of the local minimum. In fact, using a constant step size can lead to a linear convergence rate instead of a sub-linear convergence rate. To summarize, one can speed up the convergence rate at the cost of solution accuracy, which has also been reported in the recent work Pu and Nedić (2018).

In our context, we have shown the convergence of the function value sequence {*V* (Θ_*k*_)} to a neighborhood of V*. As $E\left[\omega F\left(\Theta_{k}\right)\right]\leq E\left[V\left(\Theta_{k}\right)\right]$, then we have $E\left[F\left(\Theta_{k}\right)\right]\leq \frac{1}{\omega }E\left[V\left(\Theta_{k}\right)\right]$. Since $\nabla V\left(\Theta^{*}\right)=\nabla F\left(\Theta^{*}\right)=0$, which leads to that $F^{*}=V^{*}$. It can be obtained that $E\left[F\left(\Theta_{k}\right)-F^{*}\right]\leq \frac{1}{\omega }E\left[V\left(\Theta_{k}\right)-V^{*}\right]$. Then using Theorems 1 and 2 can establish analogous convergence rate for the true value function sequence $\left\{F\left(\Theta_{k}\right)\right\}$.

Remark 3.
Let us discuss the rates of convergence suggested by Theorems 1 and 3. We observe that when the objective function is strongly convex, the function value sequence {*V*(Θ_*k*_)} can *linearly* converge to within a fixed radius of convergence, which can be calculated as follows:

$$\underset{k\to \infty }{\lim}E\left[V\left(\Theta_{k}\right)-V*\right]\leq \frac{B\left[\omega \alpha \gamma_{m}+\left(1-\omega \right)\left(1-\lambda_{N}\right)\right]}{2r_{1}\left(\omega H_{m}+\alpha^{-1}\left(1-\omega \right)\left(1-\lambda_{2}\right)\right)}.$$

When the objective function is non-convex, we cannot claim linear convergence. However, Theorem 3 asserts that the average of the second moment of the Lyapunov gradient is bounded from above. Recall that the parameter *B* bounds the variance of the “noise” due to the stochasticity of the gradient, and if *B* = 0, Theorem 3 implies that {Θ_*k*_} asymptotically converges to a first-order stationary point.

Remark 4.
For g-CDSGD, let us investigate the corner cases where *ω* → 0 or *ω* → 1. For the strongly convex case, when *ω* → 1, we have $\frac{\alpha cB}{2r_{1}}$, where $c=\frac{\gamma_{m}}{H_{m}}$ is the condition number. This suggests that if consensus is not a concern, then each iterate $\left\{\theta_{k}^{j}\right\}$ converges to its own respective $\theta_{*}^{j}$, as depicted in Figure 1. On the other hand, when *ω* → 0, the upper bound converges to $\frac{\alpha B\left(1-\lambda_{N}\right)}{2r_{1}\left(1-\lambda_{2}\right)}$. In such a scenario, each agent sufficiently communicates its own information with other agents to arrive at an agreement. In this case, the upper bound depends on the topology of the communication network. If *λ*_*N*_ ≈ 0, this results in:

$$\underset{k\to \infty }{\lim}E\left[V\left(\Theta_{k}\right)-V^{*}\right]\leq \frac{B\alpha }{2r_{1}\left(1-\lambda_{2}\right)}.$$

For the non-convex case, when *ω* → 1, the upper bound suggested by Theorem 3 is $\frac{\alpha \gamma_{m}B}{r_{1}}$, while *ω* → 0 leads to $\frac{\left(1-\lambda_{N}\right)B}{r_{1}}$, which is roughly $\frac{B}{r_{1}}$ if *λ*_*N*_ ≈ 0.

We also compare i-CDSGD and CDSGD with g-CDSGD in terms of the optimality upper bounds to arrive at a suitable lower bound for *ω*. However, due to the space limit, the analysis is presented in the Supplementary Section S1.

### 4.2 Convergence Analysis for Momentum Variants

We next provide a convergence analysis for the g-CDMSGD algorithm, summarized in the update laws given in Eq. 12. A similar analysis can be applied to i-CDMSGD. The proof techniques are developed on top of the estimate sequence method that has been applied to the centralized version (Nesterov, 2013). In the following analysis, we focus on the basic variant where $\mu =\frac{1-\sqrt{\hat{H}\alpha }}{1+\sqrt{\hat{H}\alpha }}$. Before stating the main result, we define the sequence *ϕ*_*k*_(*Θ*), *k* = 1, 2, … as:

$$\phi_{1}\left(\Theta \right)=V\left(\Theta_{1}\right)+\frac{\hat{H}}{2}\|\Theta -\Theta_{1}{\|}^{2},\;\text{and}$$

$$\phi_{k+1}=\left(1-\sqrt{\hat{H}\alpha }\right)\phi_{k}\left(\Theta \right)+\sqrt{\hat{H}\alpha }\left(\hat{V}\right.\left(y_{k}\right)+\left(S_{k},\Theta -y_{k}\right)+\frac{\hat{H}}{2}\|\Theta -y_{k}{\|}^{2}$$ (27)

where $\hat{V}$ represents the average of the objective function values of a mini-batch. We define $\phi_{k}^{*}$ as follows

$$\phi_{k}^{*}=\underset{\Theta \in R^{Nd}}{\min}\phi_{k}\left(\Theta \right)$$

Further, from Assumption 3, we see that $Var\left[S\left(y_{k}\right)\right]\leq B+B_{V}\|\nabla V\left(y_{k}\right){\|}^{2}$. Combining Assumption 4 and $\text{Var}\left[S\left(y_{k}\right)\right]≔E\left[\|S\left(y_{k}\right)-\nabla V\left(y_{k}\right){\|}^{2}\right]$, we have $E\left[\|S\left(y_{k}\right)-\nabla V\left(y_{k}\right){\|}^{2}\right]\leq B+B_{V}G^{2}$.

We now state our main result, which characterizes the performance of g-CDMSGD. To our knowledge, this is the first theoretical result for momentum-based versions of consensus-distributed SGD.

Theorem 5.
*(Convergence of g-CDMSGD, strongly convex case) Let Assumptions* 1*,* 2*,* 3*, and* 4 *hold. If the step size satisfies* $\alpha \leq \text{min}\left\{\frac{r_{1}-\left(1-\omega \right)\left(1-\lambda_{N}\right)B_{m}}{\omega B_{m}\gamma_{m}},\frac{1}{\hat{H}},\frac{1}{2\hat{\gamma }}\right\}$ *, we have:*

$$E\left[V\left(\Theta_{k}\right)-V^{*}\right]\leq {\left(1-\sqrt{\hat{H}\alpha }\right)}^{k-1}\left(\phi_{1}^{*}-V^{*}\right)+\sqrt{\frac{\alpha }{\hat{H}}}\left(B+B_{V}G^{2}\right).$$ (28)

Proof. From Lemma 5 in Supplementary Section S1., it can be obtained that

$$E\left[V\left(\Theta_{k}\right)\right]\leq E\left[\phi_{k}^{*}+\overset{k-1}{\underset{p=1}{\sum }}{\left(1-\sqrt{\hat{H}\alpha }\right)}^{k-1-p}\left\{\alpha \|S\left(y_{k}\right)-\nabla V\left(y_{k}\right){\|}^{2}\right\}\right]$$ (29)

The last inequality follows from that the coefficient $-\frac{\hat{H}}{2}\frac{1-\sqrt{\hat{H}\alpha }}{\sqrt{\hat{H}\alpha }}\leq 0$. Recalling Eq. S20 of Lemma 5 in Supplementary Section S1 and letting Θ = Θ*, and combining Eq. 29, we have

$$\begin{array}{cc} E\left[V\left(\Theta_{k}\right)\right] & \leq E\left[V^{*}+{\left(1-\sqrt{\hat{H}\alpha }\right)}^{k-1}\left(\phi_{1}^{*}-V^{*}\right)\right]\\ & +E\left[\overset{k-1}{\underset{p=1}{\sum }}{\left(1-\sqrt{\hat{H}\alpha }\right)}^{k-1-p}\left\{\alpha \|S\left(y_{k}\right)-\nabla V\left(y_{k}\right){\|}^{2}\right\}\right] \end{array}$$ (30)

As $E\left[\|S\left(y_{k}\right)-\nabla V\left(y_{k}\right){\|}^{2}\right]\leq B+B_{V}G^{2}$, therefore, the following inequality can be acquired

$$E\left[V\left(\Theta_{k}\right)-V^{*}\right]\leq {\left(1-\sqrt{\hat{H}\alpha }\right)}^{k-1}\left(\phi_{1}^{*}-V^{*}\right)+E\left[\overset{k-1}{\underset{p=1}{\sum }}{\left(1-\sqrt{\hat{H}\alpha }\right)}^{k-1-p}\left(B+B_{V}G^{2}\right)\right]$$ (31)

Using $\sum_{p=1}^{k-1}{\left(1-\sqrt{\hat{H}\alpha }\right)}^{k-1-p}\leq \sum_{t=0}^{\infty }{\left(1-\sqrt{\hat{H}\alpha }\right)}^{t}=\frac{1}{\sqrt{\hat{H}\alpha }}$ completes the proof.

Theorem 6.
*(Convergence of i-CDMSGD, strongly convex case) Let Assumptions* 1*,* 2*,* 3*, and* 4 *hold. If the step size satisfies* $\alpha \leq \text{min}\left\{\frac{r_{1}-\left(1-\lambda_{N}^{\tau }\right)B_{m}}{B_{m}\gamma_{m}},\frac{1}{\hat{H}},\frac{1}{2\hat{\gamma }}\right\}$ *, we have:*

$$E\left[V\left(\Theta_{k}\right)-V^{*}\right]\leq {\left(1-\sqrt{\hat{H}\alpha }\right)}^{k-1}\left(\phi_{1}^{*}-V^{*}\right)+\sqrt{\frac{\alpha }{\hat{H}}}\left(B+B_{V}G^{2}\right).$$ (32)

Note, although the theorem statements look the same for g-CDMSGD and i-CDMSGD, the constants $\hat{H}$ are significantly different from each other. Theorem 5 suggests that with a sufficiently small step size, using Nesterov acceleration results in a linear convergence (with parameter $1-\sqrt{\hat{H}\alpha }$) up to a neighborhood of V* of radius $\sqrt{\frac{\alpha }{\hat{H}}}\left(B+B_{V}G^{2}\right)$. When *k* → *∞*, the first term on the right hand side vanishes and substituting $\hat{H}=\omega H_{m}+\left(1-\omega \right){\left(2\alpha \right)}^{-1}\left(1-\lambda_{2}\right)$ into $\sqrt{\frac{\alpha }{\hat{H}}}\left(B+B_{V}G^{2}\right)$, we have

$$\sqrt{\frac{\alpha }{\omega H_{m}+\left(1-\omega \right){\left(2\alpha \right)}^{-1}\left(1-\lambda_{2}\right)}}\left(B+B_{V}G^{2}\right),$$

which implies that the upper bound is related to the spectral gap 1 − *λ*_2_ of the network; hence, a similar conclusion as Theorem 1 can be deduced. When *ω* → 0, the upper bound becomes $\alpha \sqrt{\frac{1}{2\left(1-\lambda \right)}}\left(B+B_{V}G^{2}\right)$. However, *ω* → 1 leads to $\sqrt{\frac{\alpha }{H_{m}}}\left(B+B_{V}G^{2}\right)$. These two scenarios demonstrates that the “gradient noise” cased by the stochastic sampling negatively affects the convergence. One can use *ω* to trade-off the consensus and optimality updates. Evidently, when compared with the non-momentum version, the upper bound is looser due to the *B*_*V*_ *G* ^2^ even when *B* = 0. However, it should be noted that *B*+ *B*_*V*_ *G* ^2^ represents the upper bound of variance of $S\left(\Theta_{k}\right)$. The analysis below shows the faster convergence rate with the cost solution accuracy.

Next, we discuss the upper bounds obtained when *k* → *∞* for g-CDSGD and g-CDMSGD. 1) *ω* → 0: When *B*_*V*_ is sufficiently small and $r_{1}\approx \frac{1}{2\sqrt{2}}$, it can be observed that the optimality bound for the Nesterov momentum variant is smaller than that for g-CDSGD as $\frac{B\alpha }{1-\lambda_{2}}\geq \frac{B\alpha }{\sqrt{1-\lambda_{2}}}$; 2) *ω* → 1: When *γ*_*m*_ and *r*_1_ are carefully selected such that $\frac{\gamma_{m}}{2r_{1}}\approx 1$, we have $B\sqrt{\frac{\alpha }{H_{m}}}\leq \frac{B\alpha }{H_{m}}$ when $\frac{\alpha }{H_{m}}>1$. Therefore, introducing the momentum can speed up the convergence rate with appropriately chosen hyperparameters.

## 5 Experimental Results

We validate our algorithms with several experimental results using the CIFAR-10 image recognition dataset (with standard training and testing sets). We have performed the experiments with different network architectures and hyperparameters. Out of several offline hyperparameters chosen, for brevity, we present results obtained with the LeNet architecture (LeCun et al., 1998). However, we note that, the behavior for other networks remain the same. Please see Supplement for results on other hyperparameters. The LeNet architecture is a convolutional neural network (CNN) (with ReLU activations) which includes 2 convolutional layers with 32 filters each followed by a max pooling layer, then 2 more convolutional layers with 64 filters each followed by another max pooling layer, and a dense layer with 512 units. The mini-batch size is set to 512, and step size is set to 0.01 in all experiments. All experiments were performed using Keras with TensorFlow (Chollet, 2015; Abadi et al., 2016). We use a sparse network topology with five agents. We use both balanced and unbalanced data sets for our experiments. In the balanced case, agents have an equal share of the entire training set. However, in the unbalanced case, agents have (randomly selected) unequal parts of the training set while making sure that each agent has at least half of the equal share amount of examples. We summarize our key experimental results in this section, with more details and results provided in the Supplementary Section 2.

**FIGURE 2:**
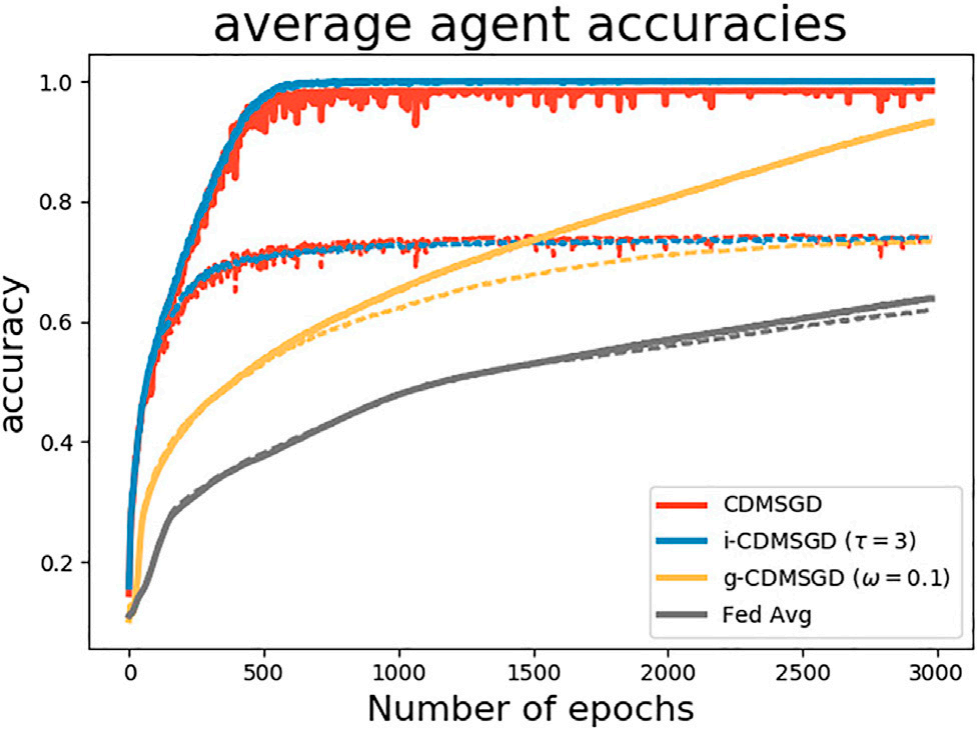
Performance of different algorithms with unbalanced sample distribution among agents (Dashed lines represent test accuracy and solid lines represent training accuracy.)

**FIGURE 3:**
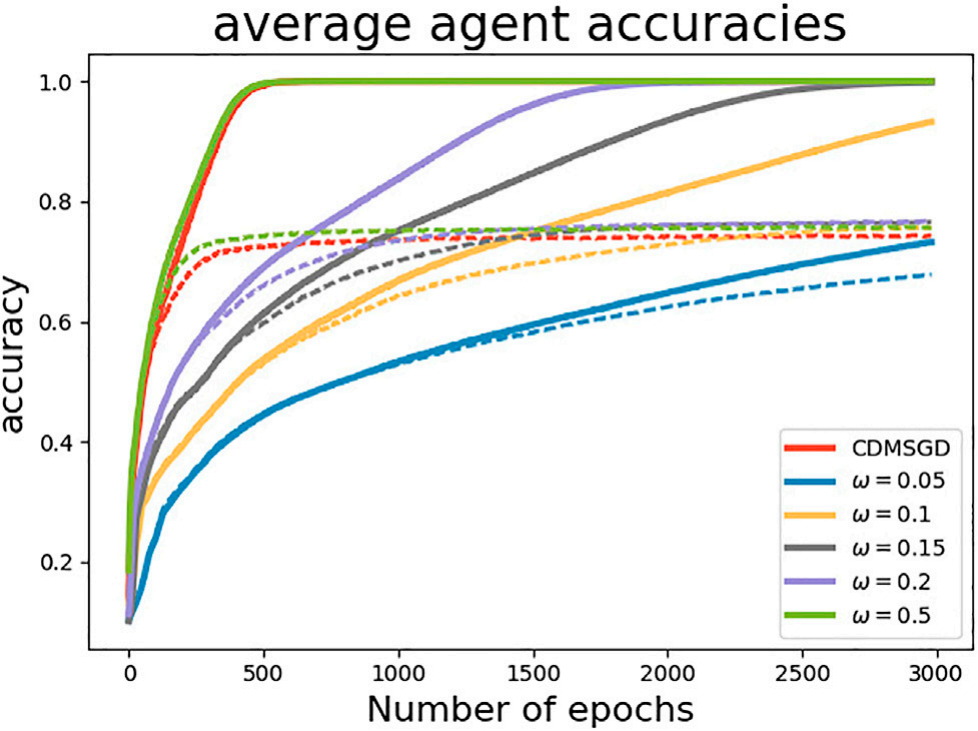
Performance of g-CDMSGD for different *ω* values (Dashed lines represent test accuracy and solid lines represent training accuracy.)

**Performance of algorithms**. In Figure 2, we compare the performance of the momentum variants of our proposed algorithms, i-CDMSGD and g-CDMSGD (with *ω* = 0.1) with state-of-the art techniques such as CDMSGD and Federated Averaging using an unbalanced data set. All algorithms were run for 3,000 epochs. Observing the average accuracy over all the agents for both training and test data, we note that i-CDMSGD can converge as fast as CDMSGD with lesser fluctuation in the performance across epochs. While being slower in convergence, g-CDMSGD achieves similar performance (with test data) with less fluctuation as well as smaller generalization gap (i.e., difference between training and testing accuracy). All algorithms significantly outperform Federated Averaging in terms of average accuracy. We also vary the tuning parameter *ω* for g-CDMSGD to show (in Figure 3) that it is able to achieve similar (or better) convergence rate as CDMSGD using higher *ω* values with some sacrifice in terms of the generalization gap.

**FIGURE 4:**
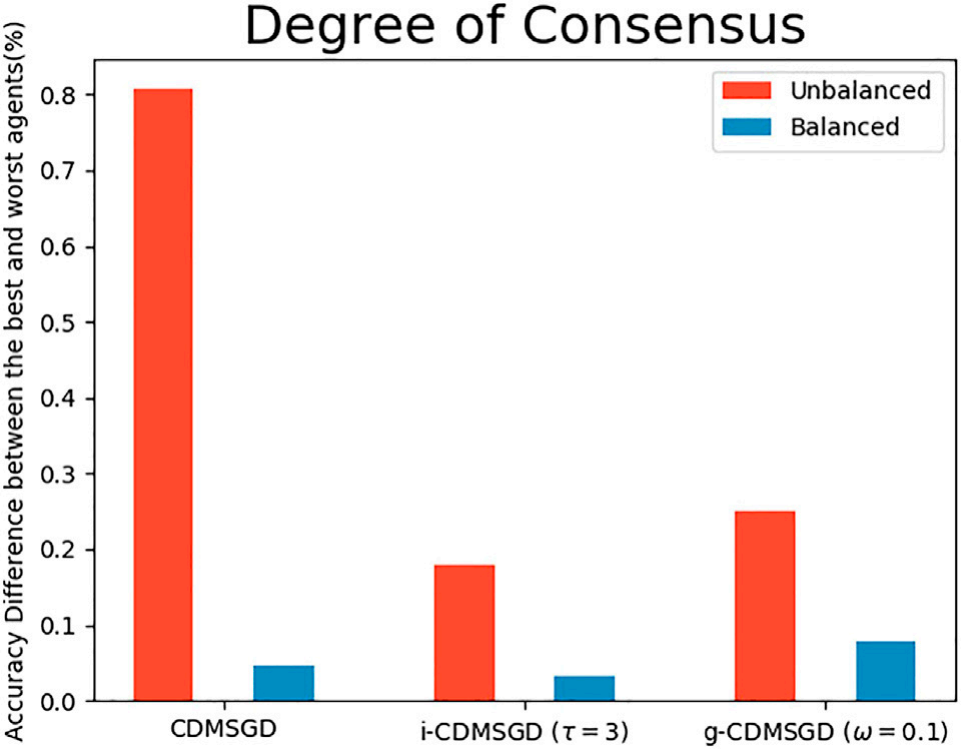
The accuracy percentage difference between the best and the worst agents for different algorithms with unbalanced and balanced sample distribution among agents.

**FIGURE 5:**
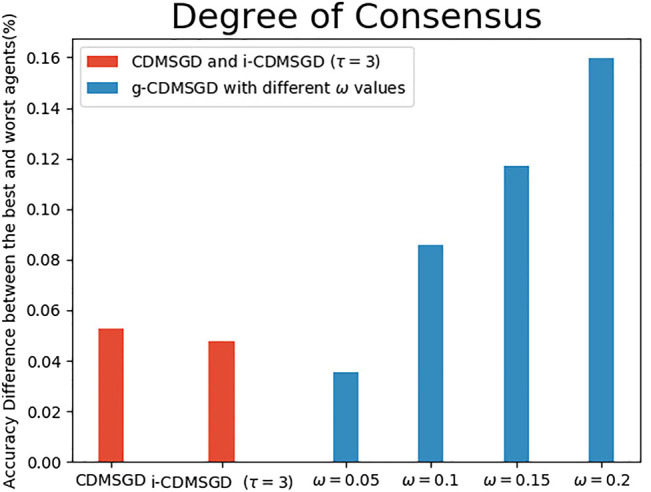
The accuracy percentage difference between the best and the worst agents with balanced sample distribution for CDMSGD, i-CDMSGD and g-CDMSGD (varying *ω*.).

**FIGURE 6:**
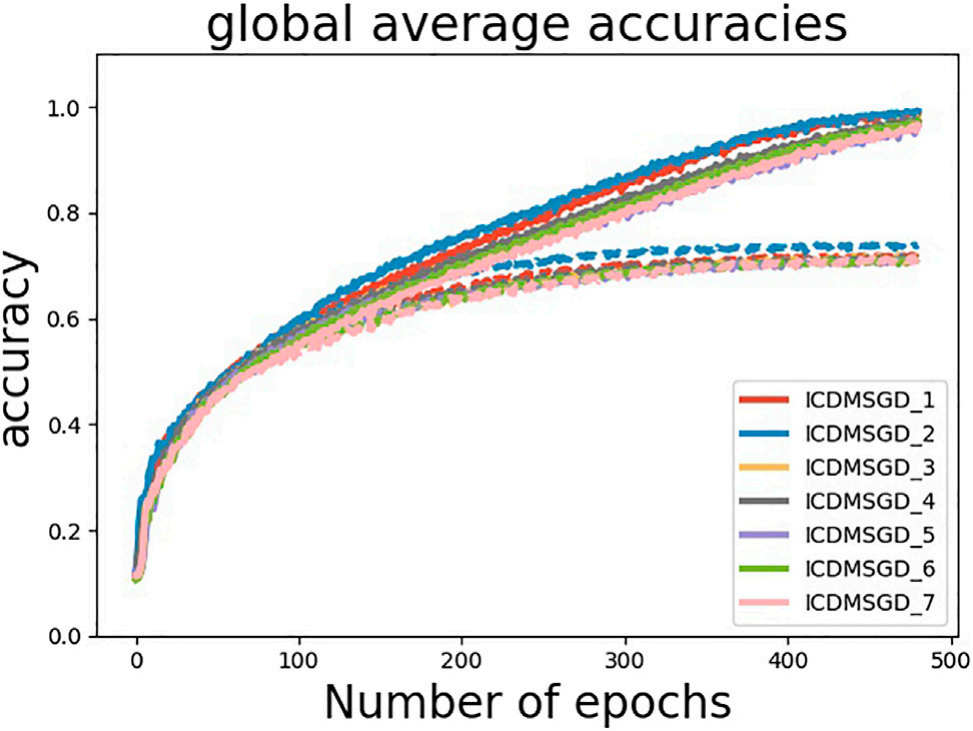
Performance of i-CDMSGD for different *τ* values (Dash lines represent test accuracy and solid lines represent training accuracy).

**Degree of Consensus**. One of the main contribution of our paper is to show that one can control the degree of consensus while maintaining average accuracy in distributed deep learning. We demonstrate this by observing the accuracy difference between the best and the worst performing agents (identified by computing the mean accuracy for the last 100 epochs). As shown in Figure 4, the degree of consensus is similar for all three algorithms for balanced data set, with i-CDMSGD performing slightly better than the rest. However, for an unbalanced set, both i-CDMSGD and g-CDMSGD perform significantly better compared to CDMSGD. Note, the degree of consensus can be further improved for g-CDMSGD using lower values of *ω* as shown in Figure 5. However, the convergence becomes relatively slower as shown in Figure 3. We do not compare these results with the Federated Averaging algorithm as it performs a brute force consensus at every epoch using centralized parameter server. In Figure 6, we show the performance of i-CDMSGD with different *τ* values. We observe that while there is better performance by increasing the value of *τ*, we see that the performance degrades after a while and then quickly stabilizes to similar performance. This is because the doubly stochastic agent interaction matrix for the small agent population becomes stationary very quickly with a very small value of *τ*. However, this will be explored in our future work with significantly bigger networks.

**FIGURE 7:**
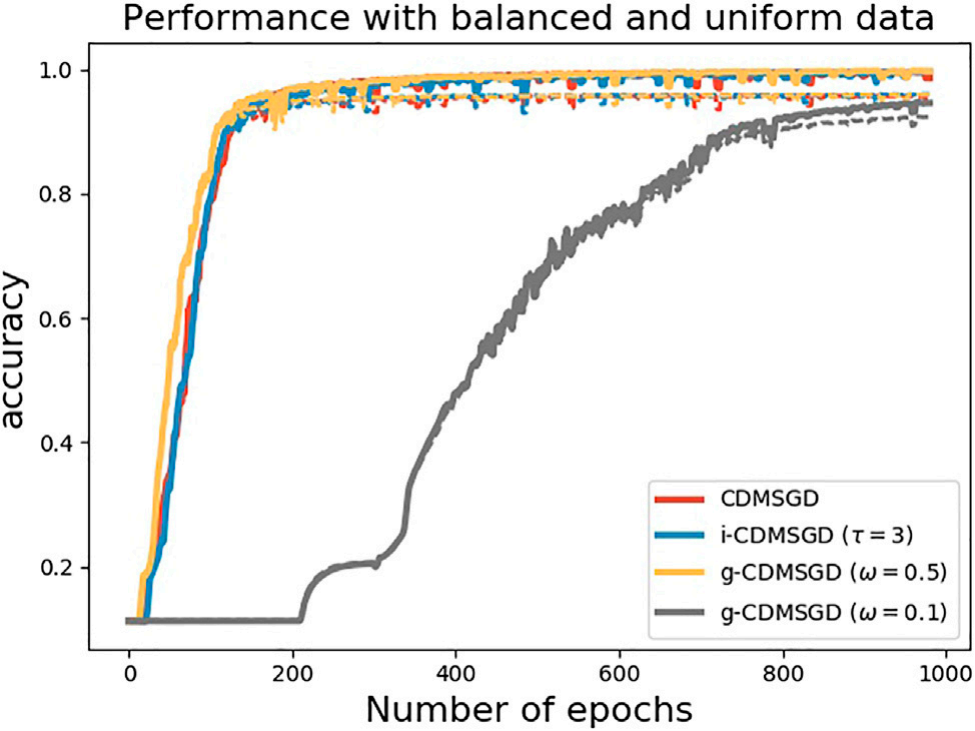
Performance of different algorithms with a balanced data distribution on MNIST dataset.

Finally, we also compare our proposed algorithms to CDMSGD on another benchmark dataset—MNIST. The performance of the algorithms is shown in Figure 7 which follows similar trend as observed for CIFAR-10.

## 6 Conclusion and Future Work

For investigating the trade-off between consensus and optimality in distributed deep learning with constrained communication topology, this paper presents two new algorithms, called i-CDSGD and g-CDSGD and their momentum variants. We show the convergence properties for the proposed algorithms and the relationships between the hyperparameters and the consensus and optimality bounds. Theoretical and experimental comparison with the state-of-the-art algorithm called CDSGD, shows that i-CDSGD, and g-CDSGD can improve the degree of consensus among the agents while maintaining the average accuracy especially when there is data imbalance among the agents. Future research directions include learning with non-uniform data distributions among agents and time-varying networks.

## Data Availability

Publicly available datasets were analyzed in this study. This data can be found here MNIST: http://yann.lecun.com/exdb/mnist/, CIFAR: https://www.cs.toronto.edu/∼kriz/cifar.html.

## Footnotes

1 As our proposed algorithm is a distributed variant of SGD, the noise in the performance is caused by the random sampling Song et al. (2015).
