# Supplementary material for: On Consensus-Optimality Trade-offs in Collaborative Deep Learning
Source: Front Artif Intell. 2021 Sep 14;4:573731. doi: 10.3389/frai.2021.573731 (PMC8478077; doi:10.3389/frai.2021.573731)
Supplement: Supplementary file 1 [file DataSheet2.PDF]

Supplementary Material

1 ADDITIONAL ANALYSIS AND PROOF

1.1 Omitted algorithms

Update rules for the momentum variant, i-CDMSGD. The compact form of i-CDMSGD is expressed as follows:

$$\mathbf{y}_{k+1} = \Theta_k + \mu(\Theta_k - \Theta_{k-1}) \tag{S1a}$$

$$\Theta_{k+1} = \mathbf{P}^{\tau}\mathbf{y}_{k+1} - \alpha\mathbf{g}(\mathbf{y}_{k+1}). \tag{S1b}$$

Rewriting the above equations yields:

$$\begin{aligned}\Theta_{k+1} &= \mathbf{y}_{k+1} - \mathbf{y}_{k+1} + \mathbf{P}^{\tau}\mathbf{y}_{k+1} - \alpha\mathbf{g}(\mathbf{y}_{k+1}) \\ &= \mathbf{y}_{k+1} - \alpha(\mathbf{g}(\mathbf{y}_{k+1}) + \frac{1}{\alpha}(I_{Nd} - \mathbf{P}^{\tau})\mathbf{y}_{k+1}).\end{aligned} \tag{S2}$$

Letting $\mathcal{S}(\mathbf{y}_{k+1}) = \mathbf{g}(\mathbf{y}_{k+1}) + \frac{1}{\alpha}(I_{Nd} - \mathbf{P}^{\tau})$, we have

$$\mathbf{y}_{k+1} = \Theta_k + \mu(\Theta_k - \Theta_{k-1}), \tag{S3a}$$

$$\Theta_{k+1} = \mathbf{y}_{k+1} - \alpha\mathcal{S}(\mathbf{y}_{k+1}). \tag{S3b}$$

1.1.1 Proofs of main lemmas and propositions

We repeat the statements of all lemmas and theorems for completeness.

Lemma 1: Let Assumptions 1 and 2 hold. The iterates of g-CDSGD (Algorithm 3) satisfy the following $\forall k \in \mathbb{N}$:

$$\mathbb{E}[V(\Theta_{k+1})] - V(\Theta_k) \leq -\alpha\nabla V(\Theta_k)^T\mathbb{E}[\mathcal{S}(\Theta_k)] + \frac{\hat{\gamma}}{2}\alpha^2\mathbb{E}[\|\mathcal{S}(\Theta_k)\|^2]. \tag{S4}$$

PROOF. By Assumption 1, the iterates generated by g-CDSGD satisfy:

$$\begin{aligned}V(\Theta_{k+1}) - V(\Theta_k) &\leq \nabla V(\Theta_k)^T(\Theta_{k+1} - \Theta_k) + \frac{1}{2}\hat{\gamma}\|\Theta_{k+1} - \Theta_k\|^2 \\ &= -\alpha\nabla V(\Theta_k)^T\nabla\mathcal{S}(\Theta_k) + \frac{1}{2}\hat{\gamma}\alpha^2\|\nabla\mathcal{S}(\mathbf{x}_k)\|^2.\end{aligned} \tag{S5}$$

Taking expectations on both sides, we can obtain

$$\mathbb{E}[V(\Theta_{k+1}) - V(\Theta_k)] \leq \mathbb{E}[-\alpha\nabla V(\Theta_k)^T\nabla\mathcal{S}(\Theta_k) + \frac{1}{2}\hat{\gamma}\alpha^2\|\nabla\mathcal{S}(\Theta_k)\|^2]. \tag{S6}$$

While $V(\Theta_k)$ is deterministic, $V(\Theta_{k+1})$ can be considered to be stochastic due to the random sampling aspect. Therefore, we have

$$\mathbb{E}[V(\Theta_{k+1})] - V(\Theta_k) \leq -\alpha\nabla V(\Theta_k)^T\mathbb{E}[\nabla\mathcal{S}(\Theta_k)] + \frac{1}{2}\hat{\gamma}\alpha^2\mathbb{E}[\|\nabla\mathcal{S}(\Theta_k)\|^2], \tag{S7}$$

which completes the proof.

Lemma 2: Let Assumptions 1, 2, and 3 hold. The iterates of i-CDSGD (Algorithm 1) satisfy the following inequality $\forall k \in \mathbb{N}$:

$$\mathbb{E}[V(\Theta_{k+1})] - V(\Theta_k) \leq -(r_1 - \frac{\hat{\gamma}}{2}\alpha B_m)\alpha\|\nabla V(\Theta_k)\|^2 + \frac{\hat{\gamma}}{2}\alpha^2 B. \tag{S8}$$

PROOF. Recalling Lemma 1 and using Assumption 2 and Remark 1, we have

$$\begin{aligned}\mathbb{E}[V(\Theta_{k+1})] - V(\Theta_k) &\leq -r_1\alpha\|\nabla V(\Theta_k)\|^2 + \frac{\hat{\gamma}}{2}\alpha^2\mathbb{E}[\|\nabla\mathcal{S}(\Theta_k)\|^2] \leq -r_1\alpha\|\nabla V(\Theta_k)\|^2 \\ &+ \frac{\hat{\gamma}}{2}\alpha^2(B + B_m\|\nabla V(\Theta_k)\|^2) = -(r_1 - \frac{\hat{\gamma}}{2}\alpha B_m)\alpha\|\nabla V(\Theta_k)\|^2 + \frac{\hat{\gamma}}{2}\alpha^2 B\end{aligned} \tag{S9}$$

which completes the proof.

Proposition 1: Let Assumptions 1, 2, 4 hold. The iterates of g-CDSGD (Algorithm 3) satisfy the following inequality $\forall k \in \mathbb{N}$, when α satisfies Eq. 15,

$$\mathbb{E}[\|\theta_k^j - s_k\|] \leq \frac{\omega\alpha\sqrt{B + B_m G^2}}{1 - \hat{\lambda}_2}, \tag{S10}$$

where $s_k = \frac{1}{N}\sum_{j=1}^N \theta_k^j$, $\hat{\lambda}_2$ is the second-largest eigenvalue of the matrix $\mathbf{Q} = (1-\omega)(\Pi \otimes I_d) + \omega I_{Nd}$.

PROOF. Rewriting the expression 6 in another form yields $\Theta_{k+1} = \mathbf{Q}\Theta_k - \omega\alpha\mathbf{g}(\Theta_k)$. Recursively applying the new form of Eq. 6 results in the following expression

$$\Theta_k = -\omega\alpha\sum_{o=0}^{k-1}\mathbf{Q}^{k-1-o}\mathbf{g}(\Theta_k) \tag{S11}$$

which follows from that the initial value of Θ_k is set 0. Let $\mathbf{s}_k = [s_k; s_k; ...; s_k] \in \mathbb{R}^{Nd}$ such that $\mathbf{s}_k = \frac{1}{Nd}(\mathbf{1}_{Nd}\mathbf{1}_{Nd}^T)\Theta_k$. Therefore, we have

$$\begin{aligned}\|\theta_k^j - s_k\| &\leq \|\Theta_k - \mathbf{s}_k\| = \|\Theta_k - \frac{1}{Nd}(\mathbf{1}_{Nd}\mathbf{1}_{Nd}^T)\Theta_k\| \\ &= \| - \omega\alpha\sum_{o=0}^{k-1}\mathbf{Q}^{k-1-o}\mathbf{g}(\Theta_o) + \omega\alpha\sum_{o=0}^{k-1}\frac{1}{Nd}(\mathbf{1}_{Nd}\mathbf{1}_{Nd}^T\mathbf{Q}^{k-1-o})\mathbf{g}(\Theta_o)\| \\ &= \| - \omega\alpha\sum_{o=0}^{k-1}\mathbf{Q}^{k-1-o}\mathbf{g}(\Theta_o) + \omega\alpha\sum_{o=0}^{k-1}\frac{1}{Nd}(\mathbf{1}_{Nd}\mathbf{1}_{Nd}^T)\mathbf{g}(\Theta_k)\| \\ &= \omega\alpha\|\sum_{o=0}^{k-1}(\mathbf{Q}^{k-1-o} - \frac{1}{Nd}\mathbf{1}_{Nd}\mathbf{1}_{Nd}^T)\mathbf{g}(\Theta_o)\| \\ &\leq \omega\alpha\sum_{o=0}^{k-1}\|\mathbf{Q}^{k-1-o} - \frac{1}{Nd}\mathbf{1}_{Nd}\mathbf{1}_{Nd}^T\|\|\mathbf{g}(\Theta_o)\| = \omega\alpha\sum_{o=0}^{k-1}\hat{\lambda}_2^{k-1-o}\|\mathbf{g}(\Theta_o)\|,\end{aligned} \tag{S12}$$

where the third equality follows from that $\frac{1}{Nd}\mathbf{1}_{Nd}\mathbf{1}_{Nd}^T\mathbf{Q} = \frac{1}{Nd}\mathbf{1}_{Nd}\mathbf{1}_{Nd}^T$, the second inequality is obtained by using Cauchy-Schwartz inequality, $\hat{\lambda}_2 < 1$.

Therefore, the following relationships can be obtained:

$$\mathbb{E}[\|\theta_k^j - s_k\|] \leq \omega\alpha\mathbb{E}[\sum_{o=0}^{k-1}\hat{\lambda}_2^{k-1-o}\|\mathbf{g}(\Theta_o)\|] = \omega\alpha\sum_{o=0}^{k-1}\hat{\lambda}_2^{k-1-o}\mathbb{E}[\|\mathbf{g}(\Theta_o)\|] \leq \frac{\omega\alpha\sqrt{B + B_m G^2}}{1-\hat{\lambda}_2}, \quad \text{(S13)}$$

which completes the proof.

Similarly, the consensus bound for i-CDSGD is shown as follows.

Proposition 2: Let Assumptions 1, 2, 4 hold. The iterates of i-CDSGD (Algorithm 1) satisfy the following inequality $\forall k \in \mathbb{N}$, when α satisfies $0 < \alpha \leq \frac{r_1 - (1-\lambda_N^\tau)B_m}{\gamma_m B_m}$:

$$\mathbb{E}[\|\theta_k^j - s_k\|] \leq \frac{\alpha\sqrt{B + B_m G^2}}{1 - \lambda_2^\tau} \quad \text{(S14)}$$

where $s_k = \frac{1}{N}\sum_{j=1}^N \theta_k^j$.

PROOF. Rewriting Eq. 5 yields $\Theta_{k+1} = \mathbf{P}^\tau\Theta_k - \alpha\mathbf{g}(\Theta_k)$. Recursively applying the new form of Eq. 5 results in the following expression:

$$\Theta_k = -\alpha\sum_{o=0}^{k-1}\mathbf{P}^{\tau(k-1-o)}\mathbf{g}(\Theta_k) \quad \text{(S15)}$$

which follows from the fact that that the initial value of Θ_k is set 0.

Let $\mathbf{s}_k = [s_k; s_k; ...; s_k] \in \mathbb{R}^{Nd}$ such that

$$\mathbf{s}_k = \frac{1}{Nd}(\mathbf{1}_{Nd}\mathbf{1}_{Nd}^T)\Theta_k.$$

Therefore, we have:

$$\begin{aligned}
\|\theta_k^j - s_k\| \leq \|\Theta_k - \mathbf{s}_k\| &= \|\Theta_k - \frac{1}{Nd}(\mathbf{1}_{Nd}\mathbf{1}_{Nd}^T)\Theta_k\| \\
&= \| - \alpha\sum_{o=0}^{k-1}\mathbf{P}^{\tau(k-1-o)}\mathbf{g}(\Theta_o) + \alpha\sum_{o=0}^{k-1}\frac{1}{Nd}(\mathbf{1}_{Nd}\mathbf{1}_{Nd}^T\mathbf{P}^{\tau(k-1-o)})\mathbf{g}(\Theta_o)\| \\
&= \| - \alpha\sum_{o=0}^{k-1}\mathbf{P}^{\tau(k-1-o)}\mathbf{g}(\Theta_o) + \alpha\sum_{o=0}^{k-1}\frac{1}{Nd}(\mathbf{1}_{Nd}\mathbf{1}_{Nd}^T)\mathbf{g}(\Theta_k)\| \\
&= \alpha\|\sum_{o=0}^{k-1}(\mathbf{P}^{\tau(k-1-o)} - \frac{1}{Nd}\mathbf{1}_{Nd}\mathbf{1}_{Nd}^T)\mathbf{g}(\Theta_o)\| \\
&\leq \alpha\sum_{o=0}^{k-1}\|\mathbf{P}^{\tau(k-1-o)} - \frac{1}{Nd}\mathbf{1}_{Nd}\mathbf{1}_{Nd}^T\|\|\mathbf{g}(\Theta_o)\| = \alpha\sum_{o=0}^{k-1}\lambda_2^{\tau(k-1-o)}\|\mathbf{g}(\Theta_o)\|,
\end{aligned} \quad \text{(S16)}$$

where the third equality follows from that $\frac{1}{N}\mathbf{1}_N\mathbf{1}_N^T\mathbf{P} = \frac{1}{N}\mathbf{1}_N\mathbf{1}_N^T$, and the second inequality is obtained by using the Cauchy-Schwartz inequality. Therefore,

$$\mathbb{E}[\|\theta_k^j - s_k\|] \leq \alpha\mathbb{E}[\sum_{o=0}^{k-1}\lambda_2^{\tau(k-1-o)}\|\mathbf{g}(\Theta_o)\|] = \alpha\sum_{o=0}^{k-1}\lambda_2^{\tau(k-1-o)}\mathbb{E}[\|\mathbf{g}(\Theta_o)\|] \leq \frac{\alpha\sqrt{B + B_m G^2}}{1-\lambda_2^\tau}. \quad \text{(S17)}$$

which completes the proof.

Define

$$v_k = \arg\min_{\Theta\in\mathbb{R}^{Nd}} \phi_k(\Theta).$$

Lemma 3: The process generated by the Eq. 27 preserves the canonical form of functions $\{\phi_k(\Theta)\}$ when $\phi_1(\Theta) = \phi_1^* + \frac{\hat{H}}{2}\|\Theta - \Theta_1\|^2$:

$$\phi_k(\Theta) = \phi_k^* + \frac{\hat{H}}{2}\|\Theta - \Theta_k\|^2 \quad \text{(S18)}$$

Lemma 4: If $\alpha \leq \min\{\frac{r_1-(1-\omega)(1-\lambda_N)B_m}{\omega B_m \gamma_m}, \frac{1}{\hat{H}}\}$, then the sequences $\{v_k\}$ and $\{v_k - \mathbf{y}_k\}$ are defined as follows:

$$v_{k+1} = (1-\sqrt{\hat{H}\alpha})v_k + \sqrt{\hat{H}\alpha}\mathbf{y}_k - \sqrt{\frac{\alpha}{\hat{H}}}\mathcal{S}(\mathbf{y}_k) \quad \text{(S19a)}$$

$$v_k - \mathbf{y}_k = \frac{1}{\sqrt{\hat{H}\alpha}}(\mathbf{y}_k - \Theta_k) \quad \text{(S19b)}$$

The proof of both Lemmas follow from (Nesterov, 2013). We also have:

Lemma 5: Let all assumptions hold. If $\alpha \leq \min\{\frac{r_1-(1-\omega)(1-\lambda_N)B_m}{\omega B_m \gamma_m}, \frac{1}{\hat{H}}, \frac{1}{2\hat{\gamma}}\}$, then for $\forall k \in \mathbb{N}$, we have:

$$\mathbb{E}[\phi_k(\Theta)] \leq V(\Theta) + (1-\sqrt{\hat{H}\alpha})^{k-1}(\phi_1(\Theta) - V(\Theta)), \quad \text{(S20)}$$

$$\begin{aligned}\mathbb{E}[V(\Theta_k)] \leq \mathbb{E}\Bigg[&\phi_k^* + \sum_{p=1}^{k-1}(1-\sqrt{\hat{H}\alpha})^{k-1-p} \\ &\Big\{-\frac{\hat{H}}{2}\frac{1-\sqrt{\hat{H}\alpha}}{\sqrt{\hat{H}\alpha}}\|\Theta_p - \mathbf{y}_p\|^2 + \alpha\|\nabla V(\mathbf{y}_p) - \mathcal{S}(\mathbf{y}_p)\|^2\Big\}\Bigg]\end{aligned} \quad \text{(S21)}$$

The proof of this lemma follows from Lemmas 3 and 4, Lemma 1 of (Nitanda, 2014), and the expressions:

$$\begin{aligned}&(\nabla V(\mathbf{y}_k), \mathcal{S}(\mathbf{y}_k)) = \frac{1}{2}(\|\nabla V(\mathbf{y}_k)\|^2 + \|\mathcal{S}(\mathbf{y}_k)\|^2 - \|\nabla V(\mathbf{y}_k) - \mathcal{S}(\mathbf{y}_k)\|^2),\\ &\|\mathcal{S}(\mathbf{y}_k)\|^2 \leq 2(\|\nabla V(\mathbf{y}_k)\|^2 + \|\nabla V(\mathbf{y}_k) - \mathcal{S}(\mathbf{y}_k)\|^2),\\ &\|\nabla V(\mathbf{y}_k)\|^2 \leq 2(\|\mathcal{S}(\mathbf{y}_k)\|^2 + \|\nabla V(\mathbf{y}_k) - \mathcal{S}(\mathbf{y}_k)\|^2).\end{aligned}$$

The last two inequalities directly follow from the triangle inequality.

1.2 Discussion on consensus and optimality trade-offs for various algorithms

As shown in Figure 1, we formally denote the consensus bound after sufficient iterations by d_1. Observe that the consensus (upper) bound is a function of the spectral properties of the underlying communication topology (specifically, proportional to $1-\lambda_2$ for g-CDSGD, or $1-\lambda_2^\tau$ for i-CDSGD). Let us consider two illustrative example communication topologies: dense ($\lambda_2 = 0.01$) and sparse ($\lambda_2 = 0.8$). We can observe that with even $\tau = 2$, i-CDSGD has a much smaller consensus bound compared to that of CDSGD for the sparse topology. However, the improvement is negligible for the dense topology. Therefore, in practice one can achieve better consensus with higher τ for sparser topologies. For g-CDSGD, as d_1 is also a function of the parameter ω, it can be seen that with an appropriately chosen ω, one can reduce the consensus bound significantly. However, the tuning of ω can affect the optimality as we discuss later in the paper. Let $h = \sqrt{B + B_m G^2}$. For i-CDSGD, the smallest consensus bound is αh when $\tau \to \infty$, which leads to a large communication cost. Considering $\frac{\omega\alpha h}{1-\hat{\lambda}_2} \leq \frac{\alpha h}{1-\lambda_2^\tau}$, we obtain the condition $\omega \leq \frac{1-\lambda_2}{2-\lambda_2-\lambda_2^\tau}$ that guarantees g-CDSGD to have a better consensus bound than i-CDSGD.

In sparse networks, i-CDSGD performs empirically better than CDSGD in terms of optimality; here we attempt to explain why our theory suggests this is the case. For completeness we also compare g-CDSGD with i-CDSGD and CDSGD.

Comparisons between i-CDSGD and g-CDSGD. We provide optimality bounds (which can be interpreted as the Euclidean distance between $\hat{\theta}$ and θ^* in Figure 1. In this context, we give the upper bound for i-CDSGD, which is

$$\lim_{k\to\infty} \mathbb{E}[V(\Theta_k) - V*] \leq \frac{B(\alpha\gamma_m + 1 - \lambda_N^\tau)}{2r_1(H_m + \alpha^{-1}(1-\lambda_2^\tau))},$$

which demonstrates that the optimality bound is related to τ. Theorem 1 shows the optimality bound of g-CDSGD is a function of ω. We discuss the comparison for the strongly convex case; the non-convex case follows from the similar analysis techniques to obtain the conclusion. Suppose the following condition holds:

$$\frac{B[\omega\alpha\gamma_m + (1-\omega)(1-\lambda_N)]}{2r_1(\omega H_m + \alpha^{-1}(1-\omega)(1-\lambda_2))} \leq \frac{B[\alpha\gamma_m + 1 - \lambda_N^\tau]}{2r_1(H_m + \alpha^{-1}(1-\lambda_2^\tau))} \tag{S22}$$

which leads to

$$\omega \geq \frac{2H_m a - b\gamma_m + (be - da)\alpha^{-1}}{2H_m(a+e) + (ad - be)\alpha^{-1} - \gamma_m(b+d)}$$

where $a = 1-\lambda_N, b = 1-\lambda_2, e = 1-\lambda_N^\tau, d = 1-\lambda_2^\tau$. Let

$$A_1 = 2H_m a - b\gamma_m + (be - da)\alpha^{-1},$$
$$A_2 = 2H_m(a+e) + (ad - be)\alpha^{-1} - \gamma_m(b+d).$$

To guarantee the lower bound is positive and less than 1, the following condition should be satisfied:

$$A_1 > 0,\ A_2 > 0,\ A_1 < A_2. \tag{S23}$$

Based on the above condition, we obtain:

$$c < \min\{2\frac{2a+e}{2b+d}, \frac{2H_m e - 2(bc - ad)\alpha^{-1}}{dH_m}\}$$

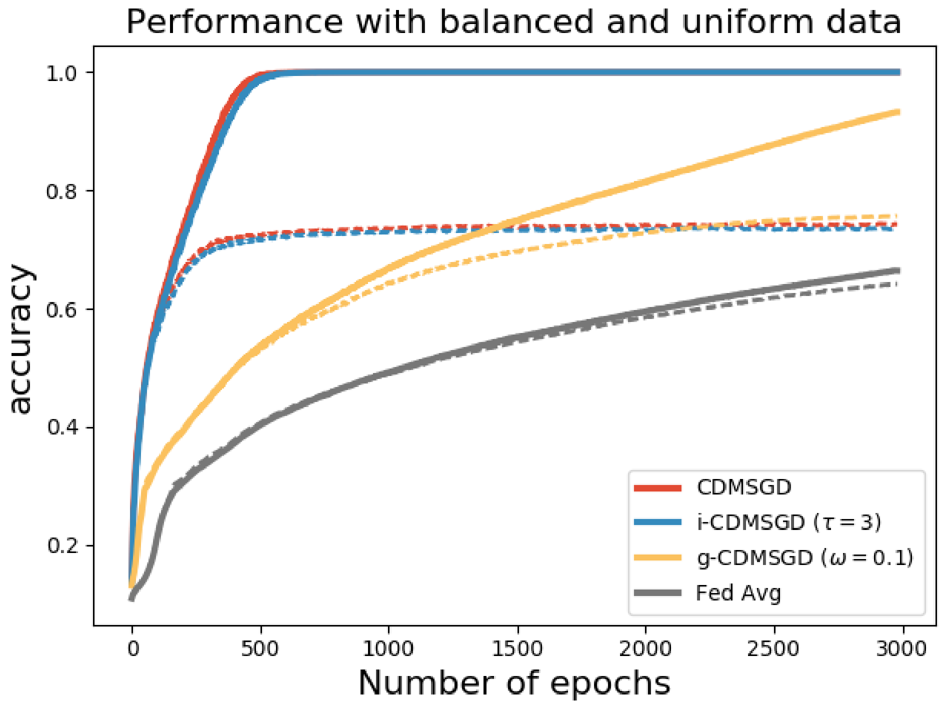

Figure S1: Performance of different algorithms on balanced and uniformly distributed data among agents. (Dashed lines represent test accuracy & solid lines represent training accuracy.)

Thus the lower bound for ω is obtained for the guarantee that g-CDSGD has a better optimal bound than i-CDSGD in strongly convex case.

Comparison between CDSGD and g-CDSGD. Given the optimality upper bound of CDSGD when $k \to \infty$ (Jiang et al., 2017) as follows:

$$\lim_{k\to\infty} \mathbb{E}[V(\Theta_k) - V*] \leq \frac{B(\alpha\gamma_m + 1 - \lambda_N)}{2r_1(H_m + \alpha^{-1}(1-\lambda_2))},$$

we have:

$$\frac{B[\omega\alpha\gamma_m + (1-\omega)(1-\lambda_N)]}{2r_1(\omega H_m + \alpha^{-1}(1-\omega)(1-\lambda_2))} \leq \frac{B[\alpha\gamma_m + 1 - \lambda_N]}{2r_1(H_m + \alpha^{-1}(1-\lambda_2))} \tag{S24}$$

After some mathematical manipulations, we can obtain the following lower bound for ω:

$$\omega \geq \frac{1}{2}.$$

Combining the lower bound for ω after comparing i-CDSGD with g-CDSGD, it can be obtained that

$$\omega \geq \max\left\{\frac{1}{2}, \frac{2H_m a - b\gamma_m + (be - da)\alpha^{-1}}{2H_m(a+e) + (ad - be)\alpha^{-1} - \gamma_m(b+d)}\right\}$$

Such a result may improve the lower bound for ω to be tighter. However, since for sparse networks, i-CDSGD outperforms CDSGD, the lower bound for ω we have shown in the main contents is an enough guarantee for improving the optimality.

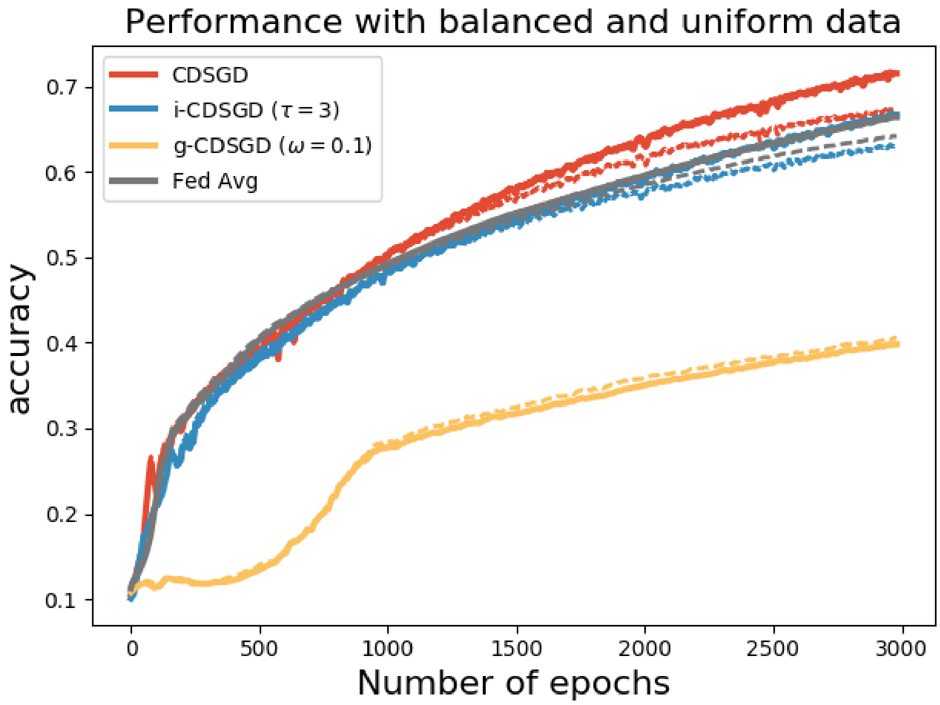

Figure S2: Performance of different non-momentum versions of the algorithms. (Dashed lines represent test accuracy & solid lines represent training accuracy.)

2 ADDITIONAL EXPERIMENTAL RESULTS

In all our experiments. we consider the number of agents to be 5. We choose the following sparse agent interaction matrix for all our experiments.

$$\pi = \begin{bmatrix} 0.34 & 0.33 & 0.0 & 0. & 0.33 \\ 0.33 & 0.34 & 0.33 & 0.0 & 0.0 \\ 0.0 & 0.33 & 0.34 & 0.33 & 0.0 \\ 0.0 & 0.0 & 0.33 & 0.34 & 0.33 \\ 0.33 & 0.0 & 0.0 & 0.33 & 0.34 \end{bmatrix}$$

More results are shown in the following figures. In Figure S1, we see that fluctuations in the average accuracy are almost negligible for the case where each agent gets balanced and uniformly distributed dataset. Algorithm i-CDMSGD performs as good as CDMSGD. We also notice that g-CDMSGD has a lower convergence rate but achieves slightly better test error which shows similar trend with Unbalanced data distribution case shown in Figure 2. Figure S2 shows the performance of the non-momentum versions of the same settings. Algorithms i-CDSGD and CDSGD perform similar to Federated Averaging whereas g-CDSGD is slow but the generalization gap is lesser.

For all the experiments until this point, each agent is allocated data from a uniform distribution of data (assured by shuffling of the data). However, it is possible that each agent can have non-uniformity in the

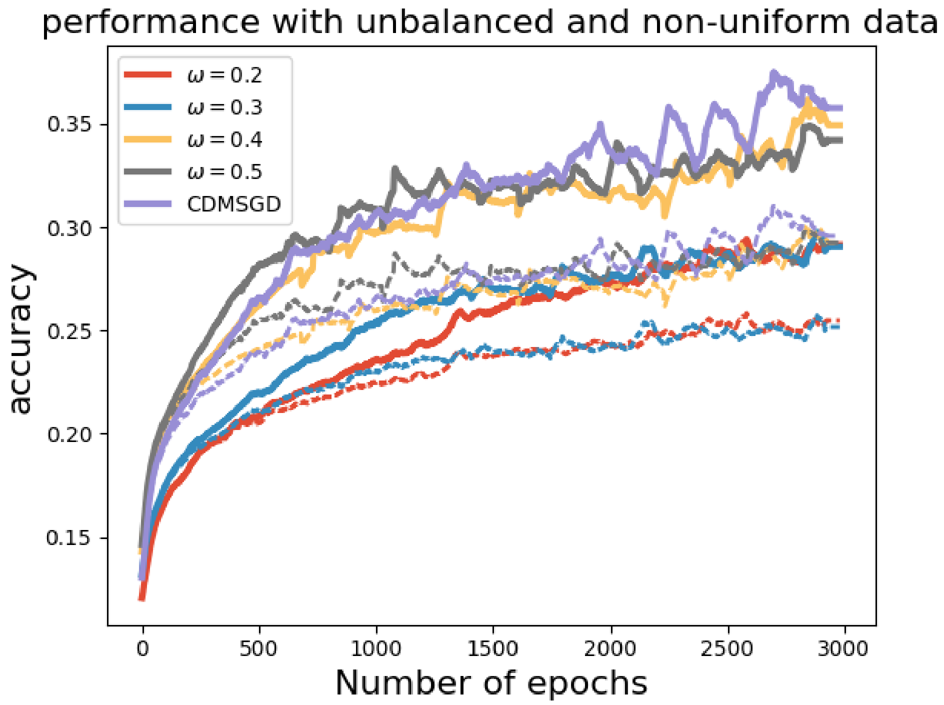

Figure S3: Performance of g-CDMSGD algorithm with different ω values with an unbalanced and non-uniform distribution of data (20% non uniformity).

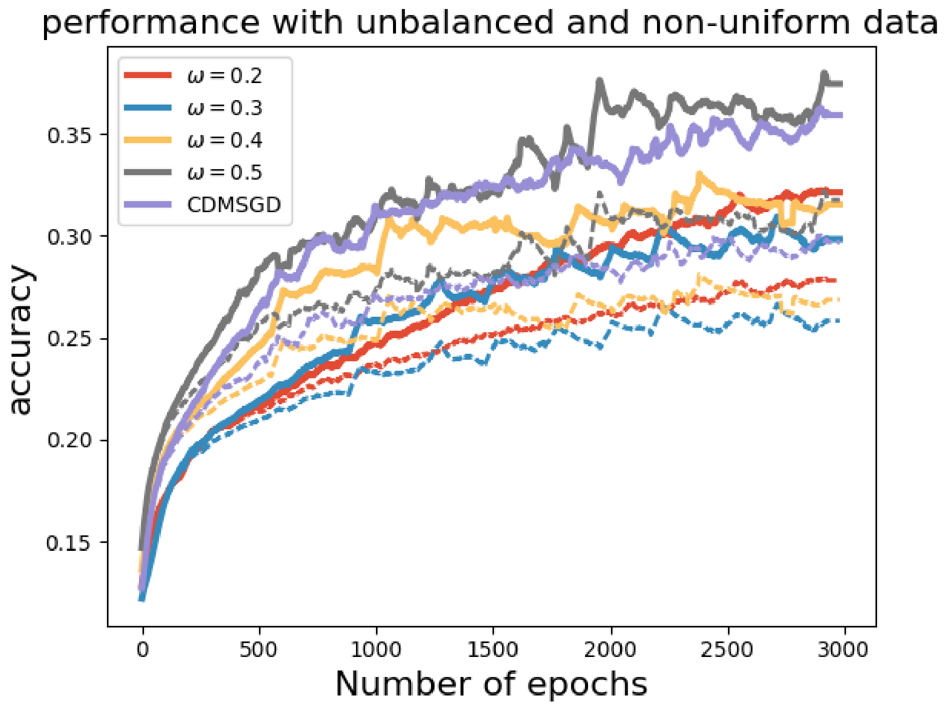

Figure S4: Performance of g-CDMSGD algorithm with different ω values with an unbalanced and non-uniform distribution of data (40% non uniformity).

distribution of data they are receiving. One of the aspect of non-uniformity is when each agent gets samples biased towards a few (not all) classes and gets very few samples of other classes. Note that this kind of distribution is referred as non-iid data distribution in Federated Averaging (McMahan et al., 2016). For simulating this, we allocate a portion of samples pertaining to a class to a specific agent and the other portion will be pooled, shuffled and distributed. Figure S3-**??** represents the performance of different algorithms with different percentage of non-uniform distribution of data (percentage of data per class allocated without any shuffling). For Figure S3, we split 20% of data pertaining to two classes to an each agent. Thus, each agent has a bias of $\approx 30\%$ towards a class. In such a non-uniform distribution of data, the performance of each agent fluctuates a lot more than the other the uniform distribution of data. With several values of ω we see that as the value of ω increases, the performance is close to CDMSGD and is even slightly better than it. At the same time, as the percentage of non-uniformity is increased to 60%, we see that the increasing ω deteriorates the performance. This can be corroborated with the increase in the agent level difference in the performance and lack of consensus as well as more emphasis on local gradient updates ($\omega = 0.5$). Since, the algorithms have not reached stability, we could not compute the degree of consensus among the agents.

REFERENCES

Jiang, Z., Balu, A., Hegde, C., and Sarkar, S. (2017). Collaborative deep learning in fixed topology networks. *Neural Information Processing Systems (NIPS)*

McMahan, H. B., Moore, E., Ramage, D., Hampson, S., et al. (2016). Communication-efficient learning of deep networks from decentralized data. *arXiv preprint arXiv:1602.05629*

Nesterov, Y. (2013). *Introductory lectures on convex optimization: A basic course*, vol. 87 (Springer Science & Business Media)

Nitanda, A. (2014). Stochastic proximal gradient descent with acceleration techniques. In *Advances in Neural Information Processing Systems*. 1574–1582
