# Supplementary figures and images for: On Consensus-Optimality Trade-offs in Collaborative Deep Learning
Source: Front Artif Intell. 2021 Sep 14;4:573731. doi: 10.3389/frai.2021.573731 (PMC8478077; doi:10.3389/frai.2021.573731)

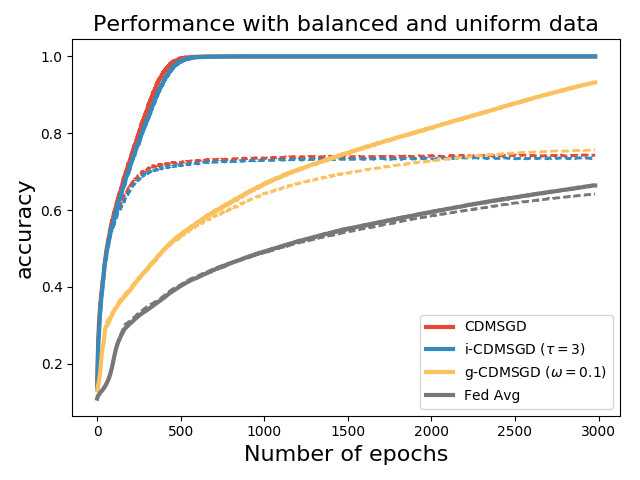

Supplement: Supplementary file 2 [file DataSheet1.ZIP › Figures for Supplementary Materials/Figure S1.jpg]

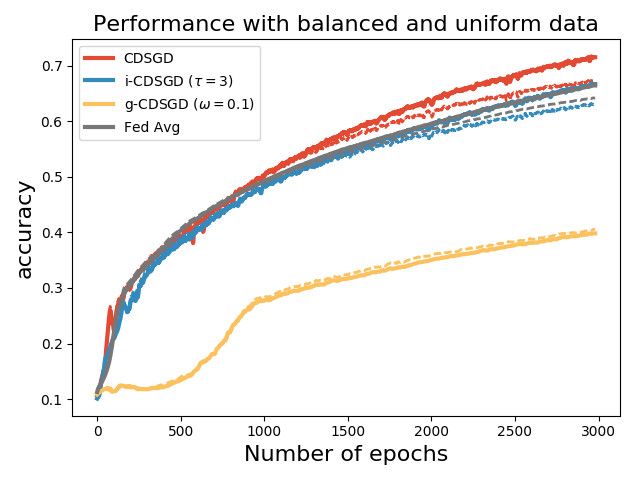

Supplement: Supplementary file 2 [file DataSheet1.ZIP › Figures for Supplementary Materials/Figure S2.jpg]

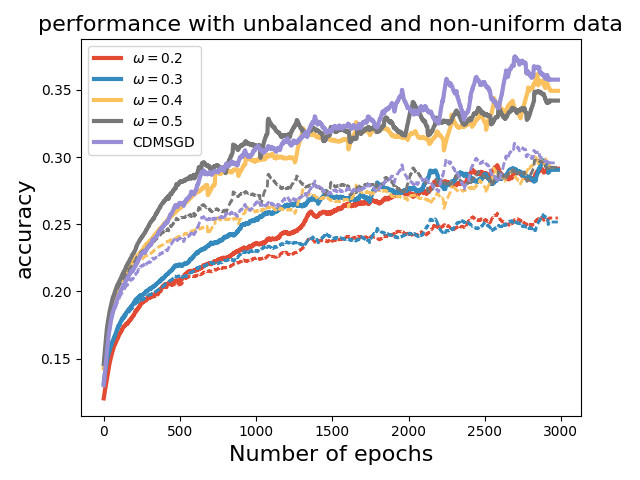

Supplement: Supplementary file 2 [file DataSheet1.ZIP › Figures for Supplementary Materials/Figure S3.jpg]

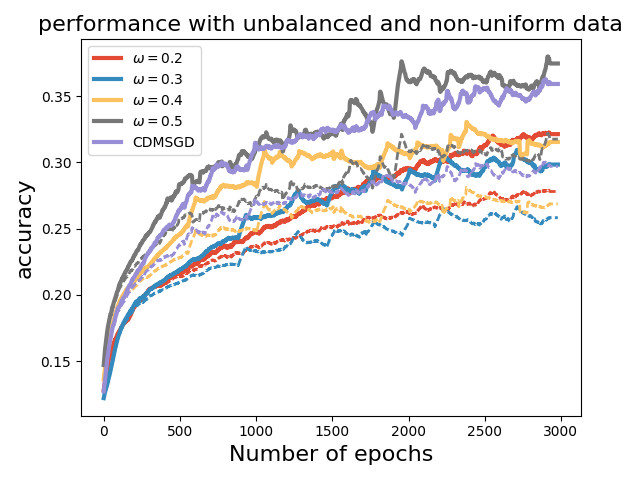

Supplement: Supplementary file 2 [file DataSheet1.ZIP › Figures for Supplementary Materials/Figure S4.jpg]

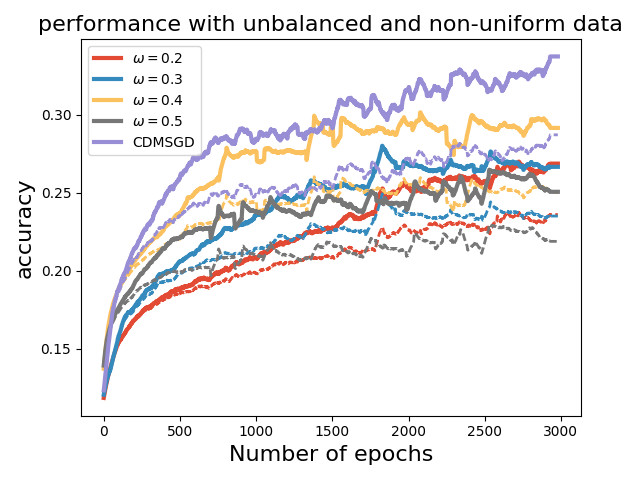

Supplement: Supplementary file 2 [file DataSheet1.ZIP › Figures for Supplementary Materials/Figure S5.jpg]

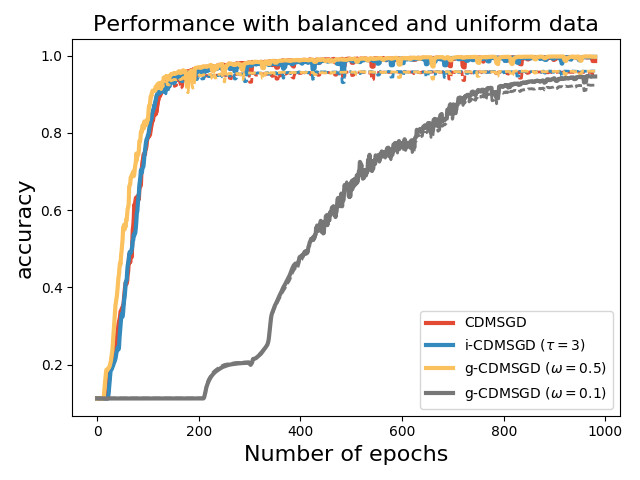

Supplement: Supplementary file 2 [file DataSheet1.ZIP › Figures for Supplementary Materials/Figure S6.jpg]
